# Genome-Scale Oscillations in DNA Methylation during Exit from Pluripotency

**DOI:** 10.1016/j.cels.2018.06.012

**Published:** 2018-07-25

**Authors:** Steffen Rulands, Heather J. Lee, Stephen J. Clark, Christof Angermueller, Sébastien A. Smallwood, Felix Krueger, Hisham Mohammed, Wendy Dean, Jennifer Nichols, Peter Rugg-Gunn, Gavin Kelsey, Oliver Stegle, Benjamin D. Simons, Wolf Reik

**Affiliations:** 1 Cavendish Laboratory, Department of Physics, JJ Thomson Avenue, University of Cambridge, Cambridge CB3 0HE, UK; 2 The Wellcome Trust/Cancer Research UK Gurdon Institute, University of Cambridge, Tennis Court Road, Cambridge CB2 1QN, UK; 3 Wellcome Trust-Medical Research Council Stem Cell Institute, University of Cambridge, Cambridge, UK; 4 Department of Physiology, Development and Neuroscience, University of Cambridge, Cambridge, UK; 5 Max Planck Institute for the Physics of Complex Systems, Noethnitzer Str. 38, 01187 Dresden, Germany; 6 Center for Systems Biology Dresden, Pfotenhauer Str. 108, 01307 Dresden, Germany; 7 Epigenetics Programme, Babraham Institute, Cambridge, UK; 8 Wellcome Trust Sanger Institute, Hinxton, Cambridge, UK; 9 School of Biomedical Sciences and Pharmacy, Faculty of Health and Medicine, The University of Newcastle, Callaghan, NSW, Australia; 10 European Molecular Biology Laboratory, European Bioinformatics Institute, Hinxton, Cambridge, UK; 11 Friedrich Miescher Institute for Biomedical Research, Maulbeerstrasse 66, 4058 Basel, Switzerland; 12 Bioinformatics Group, Babraham Institute, Cambridge, UK; 13 Centre for Trophoblast Research, University of Cambridge, Cambridge, UK

**Keywords:** pluripotency, embryo, stem cells, epigenetic, DNA methylation, dynamics, biophysical modeling

## Abstract

Pluripotency is accompanied by the erasure of parental epigenetic memory, with naïve pluripotent cells exhibiting global DNA hypomethylation both *in vitro* and *in vivo*. Exit from pluripotency and priming for differentiation into somatic lineages is associated with genome-wide *de novo* DNA methylation. We show that during this phase, co-expression of enzymes required for DNA methylation turnover, DNMT3s and TETs, promotes cell-to-cell variability in this epigenetic mark. Using a combination of single-cell sequencing and quantitative biophysical modeling, we show that this variability is associated with coherent, genome-scale oscillations in DNA methylation with an amplitude dependent on CpG density. Analysis of parallel single-cell transcriptional and epigenetic profiling provides evidence for oscillatory dynamics both *in vitro* and *in vivo*. These observations provide insights into the emergence of epigenetic heterogeneity during early embryo development, indicating that dynamic changes in DNA methylation might influence early cell fate decisions.

## Highlights

• Co-expression of DNMT3s and TETs promotes genome-scale oscillations in DNA methylation
• Oscillation amplitude is greatest at a CpG density characteristic of enhancers
• Cell synchronization reveals oscillation period and link with primary transcripts
• Multi-omic single-cell profiling provides evidence for oscillatory dynamics *in vivo*

## Graphical Abstract

**Figure:**
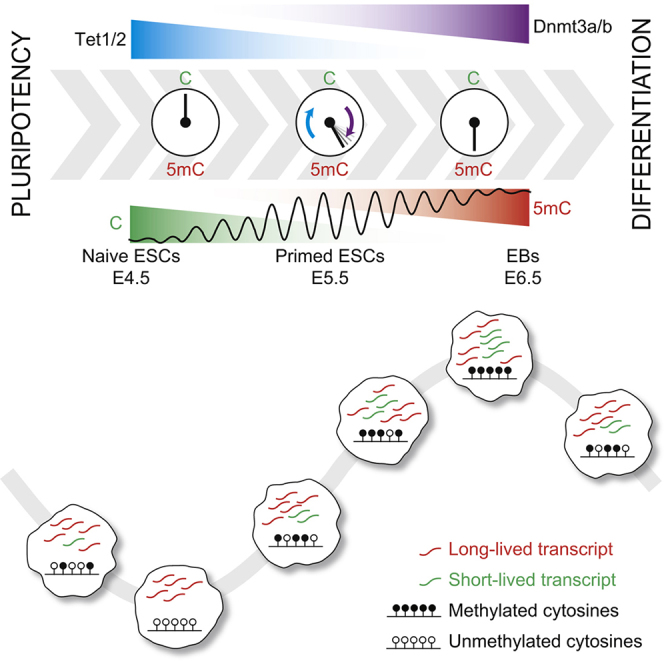

## Teaser

Exit from pluripotency and priming for differentiation into somatic lineages are associated with genome-wide *de novo* DNA methylation. We show that during this phase, co-expression of DNMT3 and TET enzymes promotes coherent, genome-scale oscillations in DNA methylation *in vitro* and *in vivo*. These oscillations are paralleled by oscillations in short-lived transcripts.

## Introduction

In mammalian embryonic development, the segregation of lineages giving rise to different somatic tissues is associated with large-scale changes in DNA methylation (5-methylcytosine). Following fertilization, global loss of DNA methylation from both the maternal and paternal genomes is tightly linked with the acquisition of naïve pluripotency in the inner cell mass of the blastocyst (Lee et al., 2014). During the transition toward the primed pluripotent state, *de novo* methylation results in a global gain of this epigenetic mark (Auclair et al., 2014, Seisenberger et al., 2012, Smith et al., 2012, Wang et al., 2014). A similar event occurs *in vitro* when embryonic stem cells (ESCs) transition from naïve to primed states, before their exit from pluripotency (Ficz et al., 2013, Habibi et al., 2013, Leitch et al., 2013, Takashima et al., 2014, von Meyenn et al., 2016). During this transition, not only are the *de novo* methyltransferases (DNMT3A/B) dramatically upregulated but the hydroxylases that initiate removal of DNA methylation (ten-eleven translocase [TET1/2]) also remain highly expressed. This paradoxical observation suggests a dynamic system, with a constant turnover of cytosine modifications (Lee et al., 2014). This could lead to the development of heterogeneous epigenetic states, with potential consequences for gene expression and cell phenotype.

DNA methylation and chromatin dynamics have been modeled quantitatively in various genomic contexts in bulk data and in exquisite detail at single loci of biological significance (Atlasi and Stunnenberg, 2017, Berry et al., 2017, Bintu et al., 2016, Haerter et al., 2014). However, the recent availability of methylome information from single-cell whole genome bisulfite sequencing (scBS-seq, Farlik et al., 2015, Smallwood et al., 2014) provides an unprecedented opportunity to study DNA methylation dynamics in the whole genome in cells undergoing a biological transition. Indeed, scBS-seq studies have already revealed profound methylation heterogeneity in ESCs, particularly in enhancers (Farlik et al., 2015, Smallwood et al., 2014).

Here, we combine single-cell sequencing with biophysical modeling to study how DNA methylation heterogeneity arises during the transition from naïve to primed pluripotency, using both *in vitro* and *in vivo* assays. We find evidence for genome-scale oscillatory dynamics of DNA methylation during this transition, with a link to primary transcripts, suggesting that heterogeneity can be created by molecular processes, not only locally but also on the genome scale.

## Results

### Heterogeneous Methylation Distributions in Primed ESCs

**Figure 1:**
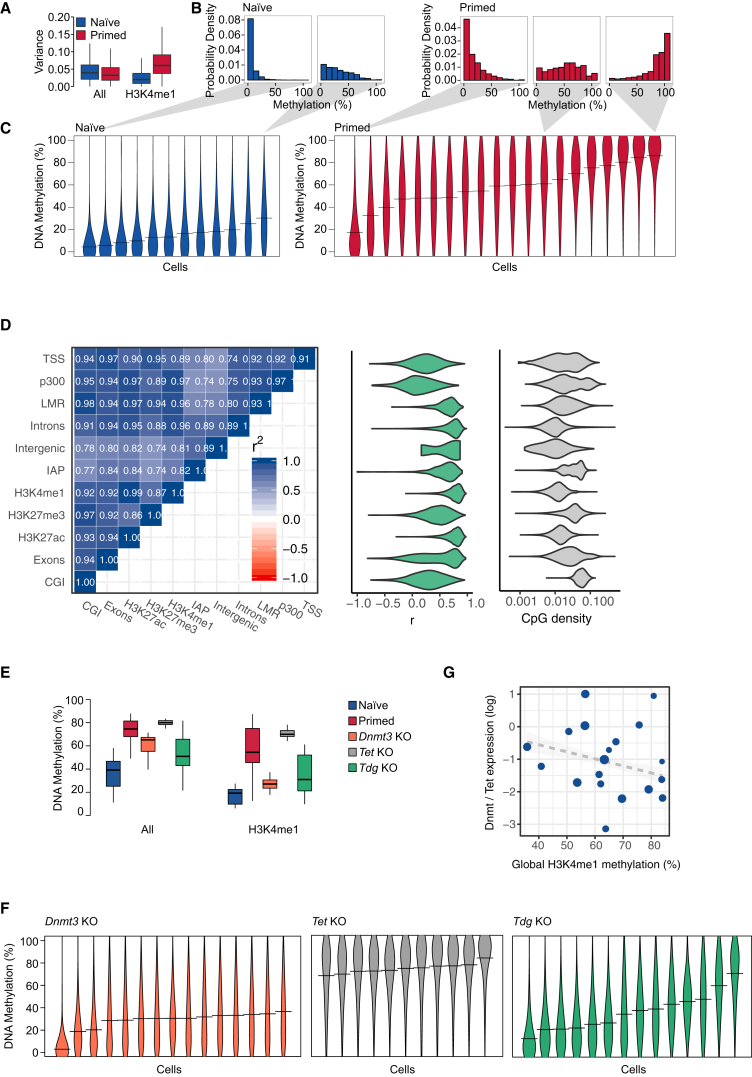
Correlated Heterogeneity in DNA Methylation (A) DNA methylation variance in naïve and primed ESCs compared for 3 kb tiles over the whole genome (All) or for enhancer elements defined using published H3K4me1 ChIP-seq data (Creyghton et al., 2010). The difference between serum and primed conditions was statistically significant (t test, p smaller than machine precision). (B and C) Analysis of DNA methylation at H3K4me1 sites in individual naïve and primed ESCs. Each histogram (B) and violin (C) represents an individual cell, and shaded triangles indicate where the same cell is shown in (B) and (C). (D) Left: Correlation heatmap for DNA methylation in different genomic features (Table S1). For every single cell, average DNA methylation levels were calculated for each genomic feature. Correlations between genomic features were then calculated using all single cell averages. (Table S1). Middle: Distribution of Pearson’s correlation coefficient between methylation levels at specific regions and global average H3K4me1 methylation. Right: Distributions of CpG densities in the same features, as defined by the number of CpGs divided by the number of base pairs. (E) Box plots showing the distribution of mean methylation rates for *Dnmt3a/b*, *Tet1-3*, or *Tdg* knockout (KO) and for wild-type ESCs in naïve and primed conditions for 1 kb tiles over the whole genome (All), or for those tiles overlapping H3K4me1 sites. Note that the effect of *Dnmt3* KO is specific to tiles overlapping H3K4me1 sites. (F) Violin plots of DNA methylation at H3K4me1 sites for individual *Dnmt3a/b*, *Tet1-3*, or *Tdg* KO ESCs cultured in primed conditions. (G) Scatter plot comparing average DNA methylation at H3K4me1 sites and transcription of DNA methylation modification enzymes in scM&T-seq data (Angermueller et al., 2016) from the “more pluripotent” sub-population of primed ESCs (see Figure S2C). Specifically, the y axis shows the sum of log-expression values of *Dnmt3* genes divided by the sum of log expression values of *Tet* genes. The size of the dots is proportional to the global methylation coverage.

To study DNA methylation during the phase of lineage priming, we began by considering ESCs, which serve as a powerful *in vitro* model for cells transiting from naïve through primed pluripotency and into early cell fate decision making (Kalkan et al., 2017). Extending previous reports (Smallwood et al., 2014), we analyzed scBS-seq data separately for ESCs cultured under naïve (“2i”) and primed (“serum”) conditions (STAR Methods). We found that primed ESCs had increased variance at several genomic annotations associated with active enhancer elements (Figures 1A and Figure S1A), including H3K4me1 and H3K27ac sites (Creyghton et al., 2010) as well as low methylated regions (LMRs) (Stadler et al., 2011). Taking published H3K4me1 chromatin immunoprecipitation sequencing (ChIP-seq) data from primed ESCs (Creyghton et al., 2010) as a broad definition of enhancer elements, we found that individual primed ESCs had average DNA methylation levels varying between 17% and 86% at enhancers (Figures 1B and 1C). Notably, single ESCs were isolated from the G0/G1 phase (Smallwood et al., 2014), suggesting that DNA methylation variance is not explained by the cell cycle stage. Correlating global DNA methylation with replication timing obtained from previously published repli-seq data (Hiratani et al., 2010) confirmed that late-replicating regions did not have lower DNA methylation than early-replicating regions (Figure S1B). In contrast to primed ESCs, naïve ESCs showed minimal cell-to-cell variability at enhancers (Figures 1B and 1C, Figures S1C and S1D), and DNA methylation heterogeneity was resolved upon differentiation to embryoid bodies (Figures S2A and S2B). This suggests that DNA methylation variance at enhancers is a unique feature of primed pluripotency. Although other genomic contexts showed proportionately less variability, levels of DNA methylation at these sites were found to be tightly correlated with those at enhancer regions and highly coherent for CpG-poor elements (Figure 1D, Figures S1A and S1C, and Table S1). DNA methylation heterogeneity in enhancer regions is, therefore, a reflection of coherent (i.e., synchronized) changes that affect DNA at the genome scale within individual cells. In the results described in this article, we will use global DNA methylation levels at enhancers as a representation of global DNA methylation in CpG-poor regions.

To assess DNA methylation heterogeneity in different transcriptional states, we used scM&T-seq to profile in parallel the methylome and transcriptome of individual ESCs (Angermueller et al., 2016). In analogy to previous work (Chambers et al., 2007, Hayashi et al., 2008, Singh et al., 2007, Toyooka et al., 2008), primed ESCs were classified into 3 subpopulations, based on hierarchical clustering of 86 pluripotency and differentiation genes (Table S2), as previously described (Figure S2C) (Kolodziejczyk et al., 2015). *Rex1*-high, “more pluripotent,” ESCs showed a wide range of average DNA methylation levels at enhancers, similar to the broad distribution seen in the primed ESC scBS-seq data (Figures 1B and 1C; Figure S2C). However, “differentiation primed” ESCs and those “on the differentiation path” had uniformly high average methylation levels at enhancer elements (Figure S2C).

Methylation of cytosine residues is catalyzed by the *de novo* DNA methyltransferase (DNMT3A/B) enzymes, while TET1/2/3 enzymes act in a multi-step process that can remove DNA methylation (Wu and Zhang, 2014). Primed ESCs express both *Dnmt3a/b* and *Tet1/2*, while naïve ESCs express *Dnmt3a/b* at much reduced levels (Figure S2B), raising the possibility that DNA methylation heterogeneity is dependent on this paradoxical co-expression (Lee et al., 2014). Consistently, we observed a loss of heterogeneity at enhancers during differentiation to embryoid bodies, where *Tet* and *Dnmt3* are downregulated (Figures S2A and S2B). Furthermore, in primed ESCs, deletion of *Dnmt3a/b* resulted in homogeneously low DNA methylation levels, while loss of *Tet1-3* led to uniformly high DNA methylation at enhancers (Figures 1E and 1F).

How does strongly correlated DNA methylation heterogeneity arise during the transition from naïve to primed pluripotency? One possibility is that methylation differences between primed ESCs reflect slow dynamic changes in the expression of DNMT3A/B and TET1/2 arising, for example, through transcriptional state switching (Singer et al., 2014). However, although DNA methylation heterogeneity is dependent on the co-expression of genes that drive methylation and demethylation (Figures 1E and 1F), analysis of scM&T-seq data (Angermueller et al., 2016) shows that global methylation levels (i.e., genome-wide mean methylation rates) are largely independent of expression (Figures 1G and S2D, R^2^ = 0.06). Moreover, DNA methylation is dynamic in primed conditions (Singer et al., 2014) and, as a system in steady-state, it follows that such dynamics must be recurrent. Such behavior could be achieved by DNA methylation switching stochastically and reversibly between distinct average levels or, alternatively, by continuously oscillating. Crucially, since we observe strong genome-wide coherence in DNA methylation levels (Figure 1D), such recurrent changes must be correlated across the genome, i.e., the methylation state of many loci must be synchronized within individual ESCs.

### Modeling Dynamics of DNA Methylation

To assess whether DNA methylation turnover could give rise to oscillatory dynamics, we turned to modeling. Notably, our approach was constrained by the genome-wide coherence of DNA methylation, which placed emphasis on us finding a description based on collective degrees of freedom. We therefore questioned how collective dynamics in DNA methylation could emerge despite the plethora of complexities that can influence methylation locally across the genome.

**Figure 2:**
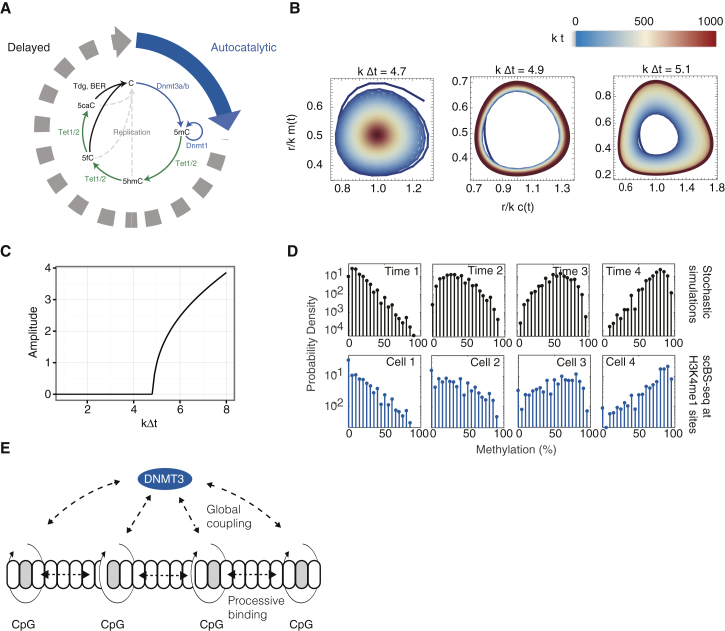
Biophysical Modeling of DNA Methylation Turnover Predicts Global Oscillations in DNA Methylation (A) Schematic summarizing the biochemical processes involved in the turnover of cytosine modifications and a biophysical model comprising autocatalytic *de novo* methylation and time-delayed demethylation. (B) Numerical solution of Equation 1 for dimensionless concentrations of (un-)methylated CpGs for various values of the dimensionless delay time kΔt (see main text and STAR Methods). Color denotes time, such that early times are blue, intermediary times are yellow, and late times are red. (C) Amplitude of oscillations (arbitrary units) as a function of the dimensionless time delay. (D) Top: Distributions of methylation rates at H3K4me1 sites as obtained from stochastic simulations. Panels show different time points. Bottom: Exemplary distributions of DNA methylation rates at H3K4me1 sites in different cells obtained from scBS-seq (Smallwood et al., 2014). (E) Schematic summarizing global and local modes of coupling of CpGs via DNMT3a/b binding.

We began by considering the dynamics of a single CpG site, which can assume different states, including an unmodified cytosine (C), a methylated cytosine (5mC), a hydroxymethylated cytosine (5hmC), and other states. Notably, the biochemistry of DNA methylation turnover involves a cyclical process: the binding and action of DNMT3A/B drives conversion of C to 5mC (Baubec et al., 2015, Jia et al., 2007), while demethylation occurs through a sequence of intermediary steps, each requiring the binding and release of enzymes, and ultimately the excision of intermediates and DNA repair or DNA replication (Figure 2A). DNMT3A/B has been shown to bind cooperatively to DNA (Emperle et al., 2014), implying that *de novo* methylation is autocatalytic. Meanwhile, the removal of DNA methylation leads effectively to a time delay, Δt, between the removal of the 5mC mark and the re-establishment of the unmodified cytosine. Given the known coupling between histone modifications, chromatin remodeling, and DNA methylation (Du et al., 2015, Iurlaro et al., 2017), it is likely that these different levels of regulation contribute to the non-linear feedback of DNA methylation on itself. Mathematically, we reasoned that the time evolution of C and 5mC concentrations, c(t) and m(t), averaged across the genome of individual ESCs, can therefore be captured by the minimal set of coupled rate equations (STAR Methods),

(Equation 1) $$\dot{c}\left(t\right)=k\;m\left(t-\Delta t\right)-r\;c\left(t\right)m\left(t\right),\;\dot{m}\left(t\right)=r\;c\left(t\right)m\left(t\right)-k\;m\left(t\right),$$

with k and r defining effective chemical conversion rates from C to 5mC and 5mC to C, respectively. If the time delay Δt is sufficiently long, *de novo* methylation of initially hypomethylated genomic regions will result in a rapid depletion of the pool of unmodified cytosines, which is then filled again due to the delayed conversion of 5mC to C. This can then lead to sustained oscillations in the levels of C and 5mC through a Hopf bifurcation (Figures 2B and 2C). Although the effective conversion rates k and r are unknown, the fact that the model predicts coherent oscillations for low values of kΔt suggests that coherent oscillations can occur under biologically relevant conditions. Indeed, distributions of methylation rates obtained from numerical simulations of the full stochastic dynamics (STAR Methods) resemble closely the experimental distributions (Figure 2D and Video S1). This suggests that DNA methylation oscillations can emerge due to the non-linearity of DNMT3 binding, even if the expression of DNMT3s and TETs remains constant over time.

Video S1. Stochastic Simulation of Oscillatory DNA Methylation Turnover, Related to Figures 2, Figure S3, and STAR Methods

Although this minimal model captures the essence of how global oscillations may emerge from the biochemistry of methylation turnover, its validity relies implicitly on a mechanism by which information on methylation levels is transported across the genome. How can such collective behavior arise, given the known heterogeneity of local factors influencing DNA methylation? To answer this question, we developed a more *ab initio* model, considering the stochastic dynamics of individual CpG sites, which, according to the biochemistry, cycle through multiple chemical states stochastically with a locus-specific rate. We hypothesized that coherent collective dynamics can emerge through the autocatalytic binding of DNMT3A/B. These enzymes can methylate multiple neighboring CpGs at the same time, leading to their effective short-range coupling (Haerter et al., 2014). At the same time, DNMT3A/B preferentially bind to 5mC, which represses active degradation of these enzymes (Sharma et al., 2011) and thereby leads to global positive feedback on DNA methylation (Figure 2E). We took both local and global feedback to be locally heterogeneous, mirroring local variations in enzyme binding affinity (conferred, for example, by different chromatin contexts).

To investigate whether locally heterogeneous interactions can, in theory, give rise to global oscillations in DNA methylation, we successively combined neighboring CpGs into larger and larger blocks characterized by an average methylation level, starting from CpG-dense regions and progressing to CpG-poor regions (Figure S3G). Repeating this coarse-graining procedure, we defined the effective DNA methylation dynamics at the genome scale (STAR Methods). Specifically, during this process, neighboring blocks of CpGs become increasingly uncoupled, such that the coarse-grained local oscillatory phase dynamics is described by a model involving the global heterogeneous coupling of oscillators (a Kuramoto model),

(Equation 2) $${\dot{\Theta }}_{i}={\tilde{\omega }}_{i}+{\tilde{\kappa }}_{i}\sum_{j}{\tilde{\kappa }}_{j}\;\sin\left(\Theta_{j}-\Theta_{i}\right),$$

with continuously varying phase coordinates, $\Theta_{i}$, indexed by position along the genome, effective intrinsic frequencies ${\tilde{\omega }}_{i}$, and couplings ${\tilde{\kappa }}_{i}$. In common with the original minimal model, the heterogeneous Kuramoto model exhibits a transition (through Hopf bifurcation) to a state involving coherent collective oscillations when the average coupling through DNMT3A/B binding, or the local heterogeneities of binding affinities, are sufficiently strong, *viz*.

(Equation 3) $${\langle \tilde{\kappa }\rangle }^{2}+\langle {\left(\tilde{\kappa }-\langle \tilde{\kappa }\rangle \right)}^{2}\rangle \;\geq \;\frac{2}{\pi G\left({\tilde{\omega }}_{0}\right)}.$$

Here, $G\left({\tilde{\omega }}_{0}\right)$ denotes the value of the probability distribution of coarse-grained oscillator frequencies taken at its maximum (see STAR Methods).

This result suggests that coherent oscillations can occur due to local and global feedback by DNMT3A/B and that this effect is enhanced by heterogeneity in DNMT3A/B binding affinities. To challenge the viability of such a mechanism, it is key to test model predictions under different perturbations of DNA methylation turnover. Fortunately, the local heterogeneity of DNMT3 binding affinities provides such perturbations throughout the genome. From the model, there emerge two key predictions of how the amplitude and frequency of oscillations changes throughout the genome. First, if oscillations are intrinsic to the DNA methylation machinery (rather than imposed by an extrinsic driving oscillator), the local frequency of oscillation should, at least transiently, be proportional to the local DNMT3A/B binding affinity. Second, if DNMT3A/B mediates global coherence in the phase of DNA methylation oscillations across the genome, the amplitude in a given genomic region will be proportional to the local rate of DNMT3A/B binding.

### Evidence for Rapid DNA Methylation Oscillations upon Serum Priming

**Figure 3:**
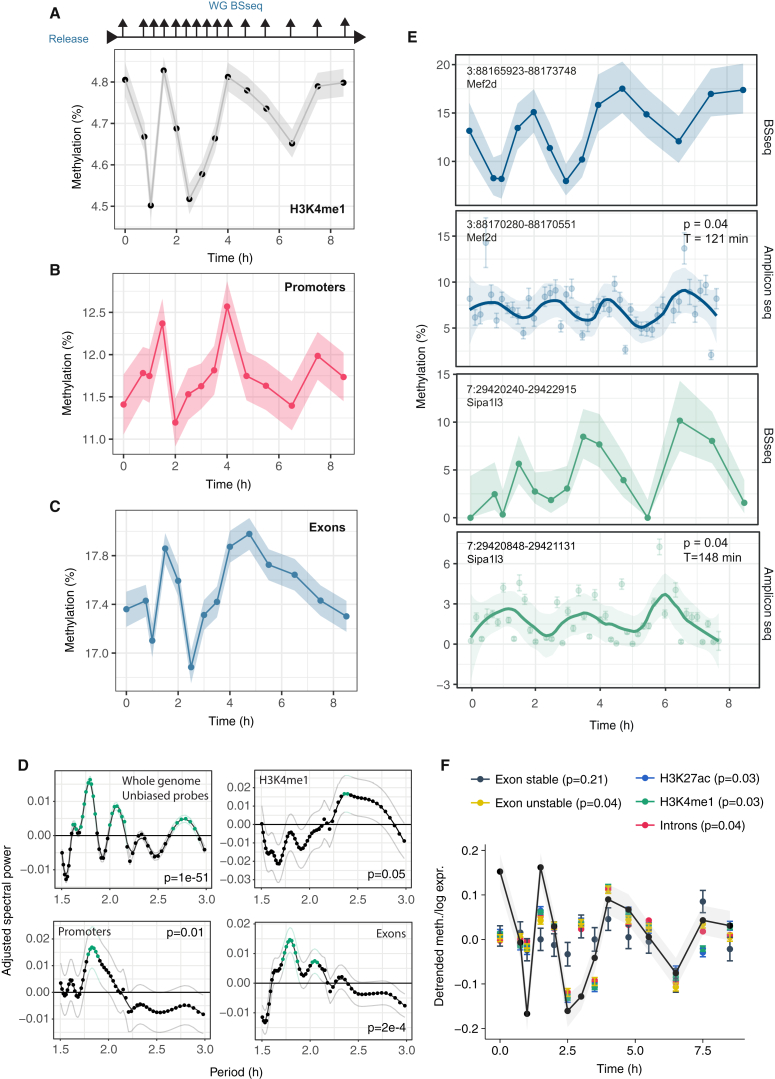
Oscillatory Dynamics of DNA Methylation during Transition from Naïve to Primed Pluripotency *In Vitro* Naïve ESCs were transferred to primed conditions in two independent “2i release” experiments. In the initial experiment, triplicate samples were collected at each time point, and BS-seq and RNA-seq libraries were prepared (A–F). In the second, single samples were collected at each time point and AmpBS-seq data are presented in (D). (A) Average DNA methylation at H3K4me1 sites over the time course. For the average, we took into account 50% of enhancers with the highest coverage depth over the time course (n = 10,324). (B) Average methylation at promoter regions (n = 637) and (C) exons (n = 4,990). (D) Average spectral densities for different genomic features calculated from whole genome BS-seq data (see also Figure S4C and STAR Methods). Green dots denote significant enrichment of a given period (p < 0.05). Thin lines denote standard errors. (E) Methylation levels at exemplary enhancer elements as measured by BS-seq (top) and AmpBS-seq (bottom). For the BS-seq experiment, dots denote methylation calls, and shaded regions signify standard errors. For the AmpBS-seq experiment, dots are methylation calls, error bars are 68% binomial confidence intervals, lines represent Loess interpolations, and shaded regions represent standard errors of the Loess interpolations. Lomb-Scargle spectral analysis was performed on the AmpBS-seq time course (STAR Methods). (F) Comparison between the DNA methylation time course in H3K4me1 regions (see also Figure 3A) and average log-expression in different genomic contexts after the removal of slow trends (STAR Methods). Shaded regions and error bars in (A)–(D) and (F) represent standard errors.

To test the model predictions and obtain more direct evidence for genome-scale DNA methylation oscillations, we next considered an *in vitro* “2i release” model in which cells were transferred from naïve 2i to primed serum culture conditions and bulk cell samples were collected for BS-seq over a subsequent time course (Figure 3A). As naïve ESCs show homogeneously low DNA methylation levels (Figure 1C), we reasoned that the transfer from naïve to primed conditions might synchronize their entry into an oscillatory phase, allowing direct evidence for oscillations to be acquired from population-based measurements. Notably, we detected evidence for rapid oscillations in the mean methylation rate over H3K4me1 enhancer domains, with a period of approximately 2–3 hr (Figure 3A and Figure S4A). Oscillations in global methylation were also observed in other genomic contexts, such as CpG-poor promoters and exons (Figures 3B, and 3C and Figures S4B and S4C). Spectral analysis confirmed enriched oscillations in H3K4me1 (p = 0.05) and H3K27ac elements (p = 0.007), as well as exons (p = 2e−4), introns (p = 8e−7), promoters (p = 0.01), and the whole genome (p = 1e−51) (Figure 3D and Figure S4C). In agreement with the model, the period of oscillations during the release differed between genomic elements (Figure 3D and Figure S4C), being longer at specific enhancer regions known to repel DNMT3 binding (Ooi et al., 2007) than at other genomic regions, such as promoters or exons. Indeed, this initial heterogeneity suggests that oscillations in DNA methylation are not driven extrinsically by a single global (genetic) oscillator, such as Hes1 (Kobayashi and Kageyama, 2011), which would lead to the same single harmonic across the genome.

At first sight, the modest amplitude of oscillations may seem to indicate that the scale of oscillatory dynamics is limited; however, global averages in bulk measurements represent only the residual signal after averaging over many noisy elements and may be confounded by cell-to-cell variability in the timing of DNMT3A/B upregulation upon priming, such that these measurements provide only a lower bound for the true local amplitude of DNA methylation oscillations. Indeed, oscillations with substantially greater amplitude were found upon inspection of specific H3K4me1 sites (Figure 3E and Figure S4D). Oscillations were yet more subtle in a repeat experiment and could not be rigorously resolved with the coverage depth available in whole-genome BS-seq. To improve our ability to detect oscillations with lower amplitudes, we designed amplicon bisulfite sequencing assays (AmpBS-seq) to target 14 loci at H3K4me1 sites that showed evidence of oscillatory dynamics in the initial 2i release experiment (Table S3). We confirmed oscillations at 4 of these 14 loci in the second experiment using spectral analysis (Figure 3D and Figure S5A), while no oscillations were observed in cells that remained in 2i (Figure S5B). Furthermore, when considering a larger set of 35 loci by AmpBS-seq (Table S1 and Figures S5A and S5B), spectral analysis revealed significantly enriched oscillations compared to control (p = 0.05, Fisher’s test).

To explore the potential impact of methylation oscillations on transcription, we performed RNA sequencing after release from 2i conditions. To resolve rapid oscillatory dynamics, we normalized reads by long-lived transcripts (Sharova et al., 2009a), which are not expected to fluctuate on short timescales. With this approach, we found significant correlations between the expression of short-lived transcripts aligned to exons, enhancer regions, and introns with global DNA methylation levels (Figure 3F).

### Oscillations Are CpG Density Dependent

**Figure 4:**
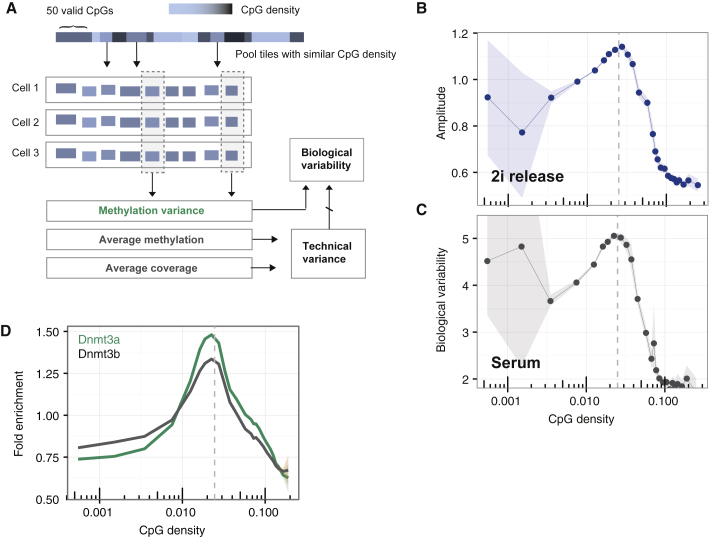
CpG Density Is a Key Parameter Defining Oscillatory Dynamics (A) Illustration of the analysis of CpG density-dependent methylation. The genome was segmented into tiles of 50 consecutive informative (i.e., at least one valid read) CpGs (unbiased probes). Regions with similar CpG densities were then grouped, and the average methylation level for a given CpG density, the variance between cells, and the average coverage in a given region were calculated. Biological variability was then determined as measured variance divided by technical variance, as an indicator of the amplitude of oscillation. (B) Amplitude of oscillations in DNA methylation following the transfer from naïve (2i) to primed conditions as a function of CpG density. Shaded regions denote 95% confidence intervals obtaind via bootstrapping. (C) Analogous analysis reveals biological variability as a function of CpG density in a long-term culture of primed ESCs (Smallwood et al., 2014). (D) Fold enrichment over input of DNMT3A/B binding as a function of CpG density. ChIP-seq data were analyzed similarly to Baubec et al. (2015). We tiled the genome into 1 kbp tiles with an overlap of 500 bp and added 8 pseudo-counts per element.

To further probe the mechanistic basis of methylation oscillations and challenge the model, we returned to the 2i release experiment to investigate whether oscillations were equally prevalent across the genome or preferentially enhanced in specific elements. The locally averaged distance between neighboring CpGs, or its inverse, the CpG density, defines a natural scale in the context of DNA methylation (Lövkvist et al., 2016). We therefore tiled the genome into windows of variable length but constant local sequencing coverage (50 informative CpGs) to account for varying CpG coverage (see Figure 4A). For each window, we then determined the CpG density and amplitude of oscillation upon 2i release, as defined by the excess variance over technical uncertainty. We found that the amplitude diverged at a characteristic value of the CpG density of around 2.5%, while oscillations were largely suppressed at CpG-rich regions (Figure 4B).

Based on this observation, we returned to the scBS-seq data for primed ESCs in steady state and calculated how much cell-to-cell variability in DNA methylation at a given region exceeds that expected from technical noise. To estimate biological variability for a given locus, and to account for confounding factors due to methylation variance, we followed previous work and considered the ratio of methylation variance across cells and the technical variance expected for a given combination of mean methylation and coverage (STAR Methods). Notably, we found the same CpG density-dependent divergence as for the amplitude of oscillations after 2i release (Figure 4C), consistent with our hypothesis that methylation heterogeneity in primed ESCs derives from oscillatory dynamics. Moreover, the divergence in the strength of oscillations coincides with the measured CpG density-dependence of DNMT3A/B binding (Figure 4D), as obtained from previous studies based on ChIP-seq measurements (Baubec et al., 2015), suggesting that, in agreement with the model, coherence is mediated through DNMT3A/B binding.

### Evidence for Coherent Oscillations in DNA Methylation *In Vivo*

**Figure 5:**
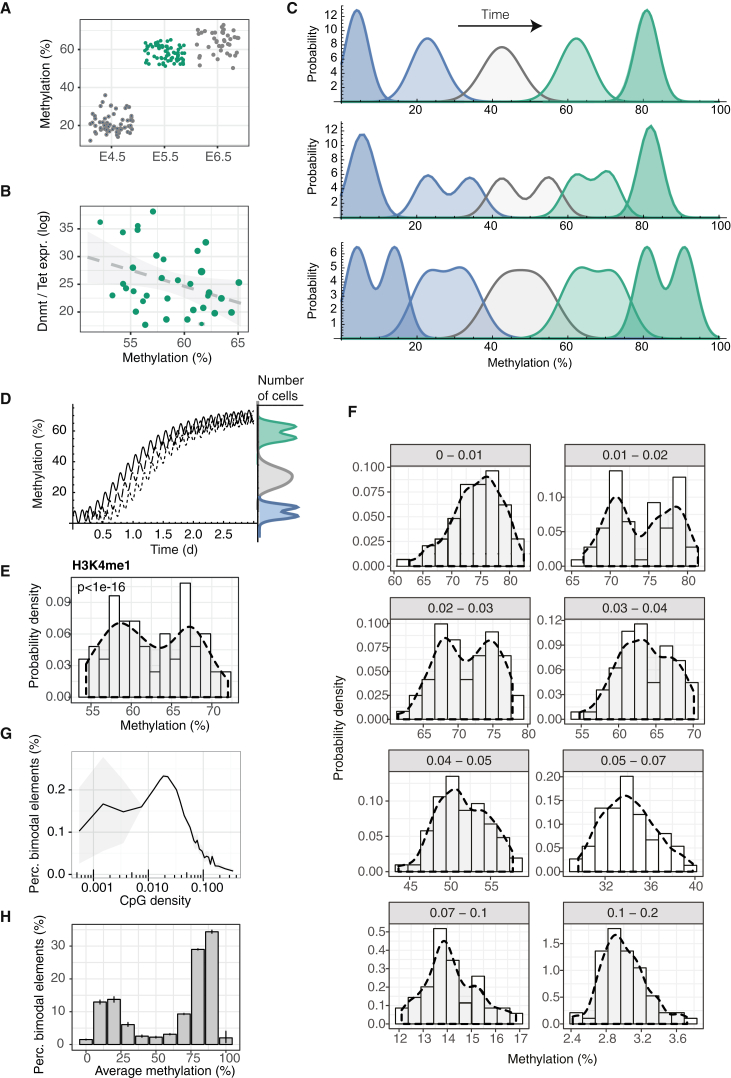
Scm&T-Seq Reveals Evidence for Oscillatory DNA Methylation *In Vivo* (A) Average DNA methylation levels at H3K4me1 sites of individual cells at three stages during early mouse embryo development (E, embryonic day). Each dot represents a cell. Error bars (standard error) are smaller than the size of the dots. (B) Average DNA methylation levels at H3K4me1 sites versus the sum of log expression levels of genes that positively influence methylation (*Dnmt3a/b/l* and *Dnmt1*) over the sum of log expression levels of genes that drive demethylation (*Tet1/2/3* and *Tdg*). The size of the dots is proportional to the overall methylation coverage; the dashed line indicates a linear fit, with shaded regions representing 95% confidence intervals. (C) Theoretical predictions of the distribution of global DNA methylation levels across cells during the stochastic process of global *de novo* methylation for various time points in different scenarios (STAR Methods). Colors denote time after exit from pluripotency, such that blue represents early times and green, late. During the process of *de novo* methylation, average methylation levels across cells (left to right) increase with time. Top: If oscillations are absent and *de novo* methylation is initiated as a unimodal distribution, the distribution of global DNA methylation levels remains unimodal at all times (or, equivalently, intermediate average methylation levels). Middle: If *de novo* methylation is initiated at early time points following a bimodal distribution (early lineage segregation), the distribution of global DNA methylation is bimodal only at intermediary times (or average methylation levels). Bottom: If global *de novo* methylation is superimposed with oscillatory dynamics, we expect bimodality at early and late times (low and high average global methylation levels) but not at intermediary times (intermediary average global methylation levels). (D) Schematic summarizing the specific patterns of the distributions of DNA methylation levels across cells *in vivo* for oscillating global DNA methylation. Biophysical modeling predicts a bimodal distribution of global methylation levels at early and late stages of global *de novo* methylation if methylation dynamics has an oscillatory component (STAR Methods). Each line represents global DNA methylation in a single cell. (E) Probability density of global DNA methylation levels across cells at H3K4me1 sites (bars: from histograms, line: density estimation) reveals evidence for bimodality at E5.5 when DNA methylation has already saturated. (F) Probability distributions (bars: from histograms, shaded areas: density estimation) of DNA methylation levels, taking into account all genomic regions but separated by different ranges of CpG densities. For this analysis, the genome was tiled into windows of 100 consecutive informative (i.e., containing at least one valid read) CpGs (unbiased probes). Bimodality is most pronounced at CpG densities that showed the largest amplitude of oscillations *in vitro* (Figure 4B). (G) Fraction of unbiased probes that show statistically significant patterns of bimodality (dip-test, p < 0.05) as a function of CpG density. (H) Fraction of unbiased probes that show statistically significant bimodality (dip-test, p < 0.05) as a function of their average methylation level across cells. Shaded regions and error bars in G and H denote 95% confidence intervals obtained via bootstrapping.

Noting that the transcriptional and epigenetic changes that occur in ESCs following their transfer from naïve to primed conditions resemble those seen *in vivo* during the exit from pluripotency (Kalkan et al., 2017), we then questioned whether oscillatory DNA methylation dynamics can be observed in the embryo. Indeed, during this transition (E4.5 to E5.5 epiblast), there is a substantial increase of *Dnmt3a* and *b* transcript levels, while *Tet1* remains highly expressed (Boroviak et al., 2015, Mohammed et al., 2017), suggesting that the co-expression of these enzymes could drive oscillations. We therefore analyzed parallel scM&T sequencing of epiblast cells at E4.5, E5.5, and E6.5 (Argelaguet et al., unpublished). Once again, we observed cell-to-cell variability in the levels of DNA methylation at primed ESC enhancers (Figure 5A and Figure S6A). At E4.5 and E6.5, global DNA methylation correlates with transcriptional changes associated, respectively, with early and late lineage priming and, in particular, Dnmt3 and Tet expression (Figures S6B and S6C). However, although DNA methylation variability is associated with transcriptional states at earlier and later stages during development (with correlations detectable using scM&T), at E5.5, as in primed ESCs, global methylation levels at enhancers were largely independent of *Dnmt3* and *Tet* expression in the same cell (Figure 5B and Figure S6B, $R^{2}=0.12$), and the transcriptome did not show any early signs of lineage priming (Mohammed et al., 2017, Peng et al., 2016). Moreover, at E5.5, DNA methylation heterogeneity was also independent of any genes that vary spatially across the embryo at E6.5 (STAR Methods) (Scialdone et al., 2016).

Based on these observations, we hypothesized that the heterogeneity of DNA methylation at E5.5 is a consequence either of stochastic *de novo* methylation or oscillatory turnover. But how can oscillatory dynamics be identified from static single-cell sequencing measures? To address this question, we reasoned that static measurements of a population of cells exhibiting oscillations around the same center point would, with higher probability, reflect cells at the extremes of the oscillation. Therefore, if the progressive increase in *de novo* methylation is superimposed with oscillatory dynamics, the distribution of average levels of DNA methylation would become bimodal, both at the onset of this transition and when DNA methylation has reached saturation (Figures 5C and 5D and STAR Methods). By contrast, during the transient phase of increasing global DNA methylation levels, cell-to-cell variability in this process would overshadow this bimodal signature, resulting in a unimodal distribution. Alternative hypotheses, such as variability in the timing of entry into the primed phase, would ultimately lead to unimodal distributions of global DNA methylation levels during the transition, with the peak tracking the increase in the average level of DNA methylation (STAR Methods).

At E5.5, when global DNA methylation levels were near their maxima, we found that the distribution of global DNA methylation across cells was indeed bimodal at enhancers (p < 1e−16) and in the whole genome (Figures 5E and 5F and Figure S5C), consistent with oscillatory dynamics. To further challenge the association of bimodality with oscillatory dynamics, we tiled the genome into coverage-based windows and used a statistical (dip) test to assess whether DNA methylation in a given window is bimodally distributed between cells. In striking agreement with the divergence of the oscillation amplitude after 2i release, and in biological cell-to-cell variability in primed conditions, the bimodal signature was strongest for elements with approximately 2.5% CpG density (Figures 5F and 5G). Further, independent of CpG density, we compared genomic regions at different stages of the *de novo* methylation process. Consistent with oscillatory dynamics (Figure 5D), and independent of CpG density, bimodality was pronounced only at hypomethylated or hypermethylated regions but not at regions with intermediary levels of methylation (Figure 5H). Notably, although our analysis does not rule out early lineage commitment through DNA methylation heterogeneity, such a scenario cannot explain the observed CpG density dependence of bimodality or the depletion of the bimodal pattern at regions with intermediary DNA methylation levels.

**Figure 6:**
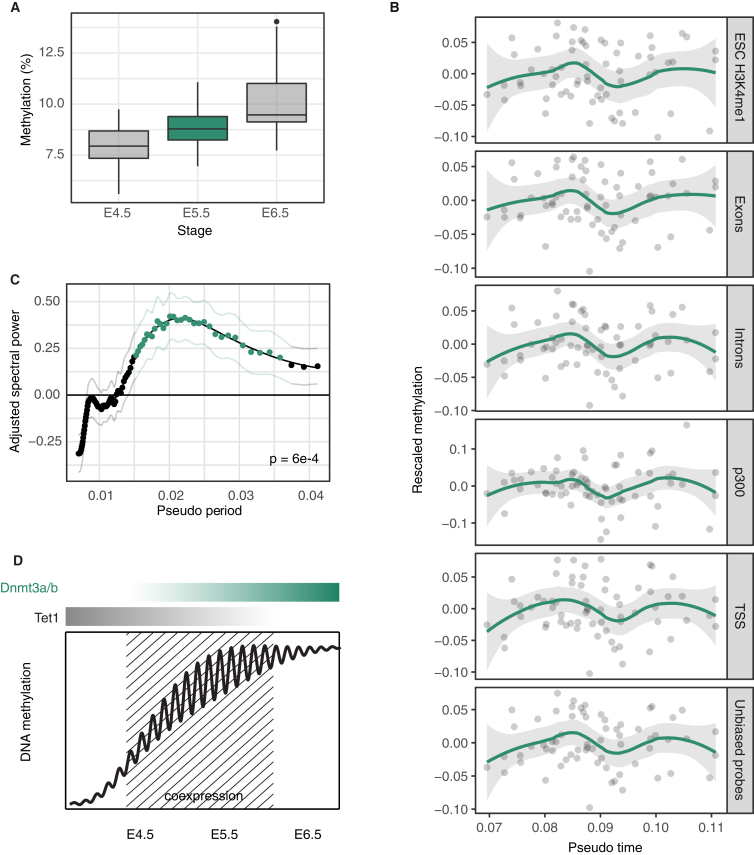
Pseudo-time Analysis Provides Independent Evidence for *In Vivo* Oscillations in DNA Methylation (A) Box plots of average DNA methylation levels of individual cells at three stages during early mouse development acquired from genomic regions with CpG densities between 10% and 15%. (B) Using the average methylation levels from (A) as a measure of the “developmental time” of a given cell, DNA methylation levels in different contexts show parallel non-monotonic dynamics. (C) Average spectral densities for the whole genome. Green dots denote significant enrichment of a given period (p < 0.05). Thin lines denote standard error. (D) Summary schematic depicting the trend for DNA methylation levels at sites of intermediate CpG density during the exit from pluripotency. As levels of DNA methylation rise during this phase, co-expression of DNMT3s and TET1 promotes intermittent genome-scale oscillations in DNA methylation.

Finally, to obtain more direct evidence for DNA methylation oscillations *in vivo*, we sought to resolve oscillations by ordering cells according to their “developmental age,” i.e., the time since the initial upregulation of the *Dnmt3* genes. To this end, we noted that CpG-rich regions do not show pronounced oscillations *in vitro*, and DNA methylation levels in these regions rise monotonically between E4.5 and E6.5 (Figure 6A). We therefore used global methylation levels in regions with a CpG density between 10% and 15% to define a methylation “pseudo-time” for individual cells (STAR Methods). Then, charting the average DNA methylation levels of genomic elements with CpG densities for which oscillations are expected to be most pronounced (i.e., between 2% and 3%) against pseudo-time, we found evidence of coherent oscillatory patterns, which were then confirmed using spectral analysis (p = 6e−4, Figures 6B and 6C; STAR Methods). In common with the findings of the 2i release experiment, this oscillatory pattern was strongest at approximately 2.5% CpG density (Figure S6D).

## Discussion

Transcriptional and epigenetic heterogeneity between cells is thought to be important for cell fate decision making during development (Torres-Padilla and Chambers, 2014), but the underlying mechanisms are largely unknown. Previous studies have estimated the degree of DNA methylation heterogeneity using single-molecule information gained from bulk BS-seq analysis (Gaidatzis et al., 2014); however, such studies cannot link patterns of heterogeneity at different genomic loci within individual cells. We have exploited single-cell sequencing to reveal genome-wide regulation of DNA methylation heterogeneity in primed pluripotent cells. By combining biophysical modeling with single-cell sequencing during exit from pluripotency, we have found evidence for DNA methylation oscillations *in vitro* and *in vivo* (Figure 6D). Mechanistically, these oscillations appear to be driven by cooperative binding of the DNMT3 enzymes, which makes low CpG density sequences, including enhancers, a particular target.

Based on the DNA modification cycle, a modeling approach predicts the emergence of genome-scale oscillations in DNA methylation when both DNMT3 and TET enzymes are expressed. These conditions arise naturally during the priming of ESCs and in epiblast cells *in vivo*, when DNMT3A/B levels increase strongly in cells already expressing TETs. By synchronizing cells in the naïve state and measuring DNA methylation upon serum priming, we found direct evidence of DNA methylation oscillations with a period of around 2–3 hr. Given the multi-step cycle of cytosine modification turnover, these oscillations are remarkably fast. However, yet more rapid oscillations in DNA methylation (with a period of 1.7 hr) were observed in breast cancer cells at the *pS2* promoter upon transcriptional activation (Métivier et al., 2008).

DNA methylation oscillations in primed ESCs are more rapid than, and therefore must be autonomous of, the cell cycle and the rate of switching between transcriptional states (Singer et al., 2014). The observation of local heterogeneity in oscillation frequency following 2i release makes it less likely that a single (genetic) oscillator, such as Hes1, which oscillates with a similar frequency, could be the driver of oscillations. However, we cannot rule out the possibility that a superposition of multiple genetic oscillators together with heterogeneous DNA binding could yield a similar phenomenology. Similarly, other factors, such as post-transcriptional changes or local chromatin dynamics, may be involved in driving oscillations.

Our initial focus was on enhancer methylation, the sites of greatest heterogeneity in primed ESCs. This is consistent with LMRs and H3K4me1 sites being targeted by hydroxymethylation in ESCs and being the most methylation-variable sequences between tissues upon differentiation *in vivo* (Booth et al., 2012, Feldmann et al., 2013a, Hon et al., 2013, Hon et al., 2014, Stadler et al., 2011). At first sight, the amplitude and genome-scale synchronization of oscillations might seem inconsistent with the limited accessibility of DNA in condensed chromatin. However, transcription factor binding to enhancers and promoters disrupts the local nucleosome structure, rendering chromatin more accessible, as reflected in DNAse hypersensitivity and ATAC-seq assays (Boyle et al., 2008, Buenrostro et al., 2013). We found that many regions of the genome participate in oscillatory methylation in a manner that is dependent on CpG density.

Parallel BS-seq and RNA-seq sequencing during 2i release suggests that oscillations in DNA methylation are correlated with changes in primary transcripts, pointing to a potential functional role. Intriguingly, in parallel with the current study, genome-scale oscillations with approximately the same period of 2–3 hr have been observed through studies of nascent transcription at intronic sites in mESCs in serum conditions (Shah et al., 2018). Through alterations in DNA binding affinities for the transcriptional machinery, mediated by changes in DNA methylation, these findings point at periodic changes in “Waddington’s epigenetic landscape” that occur on similar or faster timescales than those of cell lineage decisions. Future developments in single-cell multi-omics and the manipulation of epigenetic states *in vivo* will determine whether and how oscillations in DNA methylation play an instructive role in promoting transcriptional heterogeneity with attendant consequences for symmetry breaking and lineage priming.

## STAR★Methods

### Key Resources Table

**Table:** 

REAGENT or RESOURCE	SOURCE	IDENTIFIER
**Chemicals, Peptides, and Recombinant Proteins**		

Murine LIF	Wellcome – MRC Cambridge Stem Cell Institute	https://www.stemcells.cam.ac.uk/research/facilities/tissueculture
Mek inhibitor PD0325901	Wellcome – MRC Cambridge Stem Cell Institute	https://www.stemcells.cam.ac.uk/research/facilities/tissueculture
Gsk-3β inhibitor CHIR9902	Wellcome – MRC Cambridge Stem Cell Institute	https://www.stemcells.cam.ac.uk/research/facilities/tissueculture

**Critical Commercial Assays**		

EZ Methylation Direct MagPrep kit	Zymo	D5023
Nextera XT library prep kit	Illumina	FC-131-1096

**Deposited Data**		

Raw sequencing data	This paper	GEO: GSE75975
Published reference data	Table S1	Table S1

**Experimental Models: Cell Lines**		

Mouse: E14 embryonic stem cells	Hooper et al, (1987)	RRID: CVCL_C320 https://discovery.lifemapsc.com/stem-cell-differentiation/in-vitro-cells/inner-cell-mass-mus-musculus-e14-university-of-edinburgh
Mouse: Dnmt3a/3b embryonic stem cells	von Meyenn et al., 2016	N/A
Mouse: Tet1-3 embryonic stem cells	Hu et al., (2014)	N/A
Mouse: Tdg embryonic stem cells	Kunz et al., (2009)	N/A

**Experimental Models: Organisms/Strains**		

Mouse: C57Bl/6Babr	N/A	N/A

**Oligonucleotides**		

RT-PCR primers	Table S4	Table S4
6NF preamp oligo for (sc)BS-seq	Clark et al., (2017)	N/A
6NR adaptor 2 oligo for (sc)BS-seq	Clark et al., (2017)	N/A
PE1.0 oligo for BS-seq (PBAT)	Clark et al., (2017)	N/A
iTag indexed PE2.0 oligos	Clark et al., (2017)	N/A
iTag sequence primer	Clark et al., (2017)	N/A
Amplicon BS-seq primers	This paper	Table S3
Indexed PE1.0 oligos for amplicon BS-seq	This paper	Table S5
Biotinylated Oligo-dT30VN for single cell mRNA	Macaulay et al., (2016)	N/A
Template switching oligo (TSO) for single cell cDNA amplification	Macaulay et al., (2016)	N/A
ISPCR for single cell cDNA amplification	Macaulay et al., (2016)	N/A

**Recombinant DNA**		

N/A		N/A

**Software and Algorithms**		

Trim Galore		www.bioinformatics.babraham.ac.uk/projects/trim_galore/
Cutadapt	Martin, (2011)	https://cutadapt.readthedocs.io/en/stable/#
Bismark	Krueger and Andrews, (2011)	https://www.bioinformatics.babraham.ac.uk/projects/bismark/
SeqMonk		https://www.bioinformatics.babraham.ac.uk/projects/seqmonk/
R		https://www.r-project.org/
Perl		https://www.perl.org/

### Contact for Reagent and Resource Sharing

Further information and requests for resources and reagents should be directed to and will be fulfilled by the Lead Contact, Wolf Reik (wolf.reik@babraham.ac.uk).

### Experimental Model and Subject Details

#### Cell Lines

Mouse E14 ESCs (male) (Hooper et al., 1987) were cultured in naïve or primed conditions as described below. *Dnmt3a/b* knock-out ESCs (male) (von Meyenn et al., 2016), *Tet1-3* knock-out ESCs (male) (Hu et al., 2014) and *Tdg* knock-out ESCs (male) (Kunz et al., 2009) were grown in primed conditions as described below.

Naïve ESCs were cultured in serum free media with 2i inhibitors (Neurobasal, N2, B27, 103 U/ml LIF, 1 μM Mek inhibitor PD0325901 and 3 μM Gsk-3β inhibitor CHIR99021) without feeders at 37°C and 5% CO_2_.

Primed ESCs were cultured in serum containing media (DMEM 4,500 mg/l glucose, 4 mM L-glutamine, 110 mg/l sodium pyruvate, 15% fetal bovine serum, 1 U/ml penicillin, 1 μg/ml streptomycin, 0.1 mM nonessential amino acids, 50 μM β-mercaptoethanol, and 103 U/ml LIF ESGRO) without feeders at 37°C and 5% CO_2_.

For the 2i release experiment, ESCs in naïve culture conditions were washed with PBS before addition of media for primed ESC growth.

EB differentiation was performed by seeding 1000 primed ESCs per well in ultra-low attachment 96-well plates (Sigma-Aldrich) in primed ESC culture media without Lif.

#### Mice

All animal procedures were performed in accordance with the local ethical review committee and under license from the Animal Scientific Procedures Act 1986 (HO Project Licenses PPL70/8276). C57Bl/6Babr embryos were collected at E4.5 (n = 91 from 4 embryos), E5.5 (n = 80 from 1 embryo) and E6.5 (n = 96 from 2 embryos). The sex of embryos was not recorded at the time of collection because of their early developmental stage.

### Method Details

#### Cell Lysis and Nucleic Acid Purification

For the 2i release experiment, cells were lysed by removing media from culture dishes and adding 200ul of RLT plus buffer (Qiagen) supplemented with 0.5mM 2-mercaptoethanol. In the first experiment, triplicate samples were collected at 31 time points from 0 h to 56 h 30 min. In the second experiment, single samples were collected at 47 time points from 0 h to 7 h 40 min (every 20 min). The second experiment also included a control time-course, where cells received fresh naïve culture medium rather than primed culture medium at the beginning of the experiment. Total nucleic acid was purified from cell lysates with RNAdvance magnetic beads (Beckman Coulter, A32649) using a Bravo Workstation pipetting robot (Agilent Technologies) following the manufacturers protocol. RNA was subsequently purified by treating the total nucleic acid with DNase I.

For qPCR analysis during EB differentiation, RNA was prepared from frozen cell pellets using DNA/RNA AllPrep kits (Qiagen).

##### RT-PCR

Purified RNA was reverse-transcribed using RevertAid (ThermoFisher, EP0441) and random hexamer primers. qPCRs were performed in triplicate using Brilliant III SYBR (Agilent Technologies) or Platinum SYBR (ThermoFisher). Primer sequences are provided in Table S4.

##### FACS

FACS collection of single *Dnmt3a/b*, *Tet1-3* and *Tdg* knok-out ESCs and EB cells was performed selecting for live cells and low DNA content (i.e., G0 or G1 phase cells) using ToPro-3 and Hoechst 33342 staining.

##### Isolation of Mouse Embryonic Cells

C57Bl/6Babr E4.5 embryos were dissected from nascent decidua and trophectoderm removed by immunosurgery (Solter and Knowles, 1975). The embryos at E4.5 and E5.5 were then dissociated using Accutase™ (5 min), transferred to M2 droplets and then single cells were picked and transferred to cell lysis buffer using a finely drawn Pasteur pipette and frozen immediately. The visceral endoderm and extraembryonic ectoderm at E5.5 were both separated from the epiblast by pulling using a finely drawn Pasteur pipette. The extra embryonic ectoderm was removed using the tip of a pulled Pasteur pipette. At E6.5, embryos were dissected from decidua in PBS and placed into droplets of M2 for manual dissection to remove extraembryonic tissue. Cells were again dissociated using Accutase™ at room temperature and single cells picked, lysed and frozen as described above.

##### Bisulfite Sequencing

For the 2i release experiment, bisulfite sequencing (BS-seq) libraries were prepared from the total nucleic acid using the bulk-cell PBAT method previously described (Smallwood et al., 2014). Briefly, bisulfite conversion and purification was carried out using the EZ Methylation Direct MagPrep kit (Zymo), following the manufacturers’ instructions but with half volumes. Bisulfite converted DNA was eluted from MagBeads directly into 39ul of first strand synthesis reaction mastermix (1x Blue Buffer (Enzymatics), 0.4mM dNTP mix (Roche), 0.4uM 6NF preamp oligo (IDT) then heated to 65°C for 3 minutes and cooled on ice. 50U of klenow exo- (Enzymatics) was added and the mixture incubated on a thermocycler at 37°C for 30 minutes after slowly ramping from 4°C. Reactions were diluted to 100μl and 20U of exonuclease I (NEB) added and incubated at 37°C before purification using a 0.8:1 ratio of AMPure XP beads. Purified products were resuspended in 50μl of second strand mastermix (1x Blue Buffer (Enzymatics), 0.4mM dNTP mix (Roche), 0.4uM 6NR adaptor 2 oligo (IDT) then heated to 98°C for 2 minutes and cooled on ice. 50U of klenow exo- (Enzymatics) was added and the mixture incubated on a thermocycler at 37°C for 90 minutes after slowly ramping from 4°C. Second strand products were purified using a 0.8:1 ratio of AMPure XP beads and resuspended in 50μl of PCR mastermix (1x KAPA HiFi Readymix, 0.2uM PE1.0 primer, 0.2uM iTAG index primer) and amplified with 9 cycles. The final libraries were purified using a 0.8:1 volumetric ratio of AMPure XP beads before pooling and sequencing. All libraries were prepared in parallel with the pre-PCR purification steps carried out using a Bravo Workstation pipetting robot (Agilent Technologies). 9-12 libraries were sequenced as a multi-plex on one Illumina HiSeq 2000 lane using 125bp paired-end read length.

##### Amplicon Bisulfite Sequencing

To obtain increased sequencing depth, amplicon bisulphite sequencing (AmpBS-seq) libraries were prepared from the second 2i release experiment. Regions of interest where chosen from exemplary enhancers showing oscillatory dynamics correlated to the global H3K4me1 trends in the BS-seq data from the first 2i release experiment. Total nucleic acid isolated from the 2i release experiment underwent bisulfite conversion using Zymo reagents as described above. KAPA HiFi Uracil+ Master Mix (Kapa Biosystems) was used to amplify regions of interest using 30nM primers (Table S3), with the reverse primer including an 8N unique molecular identifier (UMI). The PCR program was: 95C 5min; 35 repeats of 98C 20s, 60C 15s, 72C 60s; 72C 10min. Amplicons were pooled for each sample and purified using Ampure XP beads (Agencourt), before a second round of PCR was used to incorporate Illumina Adaptor sequences and index samples. The PCR reaction included 11ul pooled amplicons, 200nM indexed PE1.0 (Table S5) and iPCRTag primers (Quail et al., 2011), and KAPA HiFi Master Mix (Kapa Biosystems). The PCR program was: 98C 45s; 5 repeats of 98C 15s, 65C 30s, 72C 30s; 72C 5min. Samples were then pooled and purified before library QC and sequencing was performed with up to 144 samples included on a 150bp paired-end MiSeq run.

##### Single-Cell Sequencing

Single cell BS-seq libraries were prepared from knock-out ESCs and EBs as described (Angermueller et al., 2016, Clark et al., 2017). Briefly, single-cells were lysed in 2.5ul of RLT plus buffer (Qiagen) then diluted to 10ul prior to bisulfite conversion and purification was carried out using the EZ Methylation Direct MagPrep kit (Zymo), following the manufacturers’ instructions but with half volumes. Bisulfite converted DNA was eluted from MagBeads directly into 39ul of first strand synthesis reaction mastermix (1x Blue Buffer (Enzymatics), 0.4mM dNTP mix (Roche), 0.4uM 6NF oligo (IDT) then heated to 65°C for 3 minutes and cooled on ice. 50U of klenow exo- (Enzymatics) was added and the mixture incubated on a thermocycler at 37°C for 30 minutes after slowly ramping from 4°C. First strand synthesis was repeated 4 more times with the addition of 0.25 μl of reaction mixture (1x blue buffer, 0.25mM dNTPs, 10mM 6NF preamp oligo and 25U klenow exo-). Reactions were diluted to 100μl and 20U of exonuclease I (NEB) added and incubated at 37°C before purification using a 0.8:1 ratio of AMPure XP beads. Purified products were resuspended in 50μl of second strand mastermix (1x Blue Buffer (Enzymatics), 0.4mM dNTP mix (Roche), 0.4uM 6NR adaptor 2 oligo (IDT) then heated to 98°C for 2 minutes and cooled on ice. 50U of klenow exo- (Enzymatics) was added and the mixture incubated on a thermocycler at 37°C for 90 minutes after slowly ramping from 4°C. Second strand products were purified using a 0.8:1 ratio of AMPure XP beads and resuspended in 50μl of PCR mastermix (1x KAPA HiFi Readymix, 0.2uM PE1.0 primer, 0.2uM iTAG index primer) and amplified with 12 cycles. Finally, scBS-seq libraries were purified using a 0.8:1 volumetric ratio of AMPure XP beads before pooling and sequencing. Fifteen single cell libraries plus one negative control were multiplexed together on one Illumina HiSeq 2000 lane using 125bp paired end reads.

Single-cell methylome and transcriptome libraries were prepared from embryos as previously described (Angermueller et al., 2016).

##### RNA-Seq

cDNA was prepared from 1ul of purified RNA following the Smart-seq2 protocol (Picelli et al., 2014) with 10 cycles of amplification. Nextera XT libraries were prepared as described (Picelli et al., 2014) but with one-fifth volumes and 200pg of input cDNA. Libraries were sequenced as a multiplexed pool on one Illumina HiSeq 2000 lane using 50bp single-end read length.

##### Derivation of a Phenomenological Model

The modeling approach was to, instead of attempting to precisely model every aspect of the methylation turnover cycle, define the simplest model that captures the essence of the dynamics, without making detailed assumptions about the underlying biochemical processes. This approach is justified by the observation of genome-wide coherence of DNA methylation levels (Figure 1C), constraining the dynamics to a single degree of freedom (the phase), and ruling out the possibility that existing dynamic local heterogeneities affect the global dynamics. The strategy therefore was to derive a phenomenological model for the global DNA methylation dynamics, and then in a second step justify the emergence of coherence from the local dynamics at single cytosines. To this end, the number of unmodified cytosines, and the number of methylated cytosines in a given large region of the DNA of size N at a given time, t, were defined as C(t) and M(t), respectively. While the specific choice of N is irrelevant for the following analysis it could, for example, denote the number of base pairs in the genome or the volume of the nucleus. If N is large, continuous concentrations may be defined as c(t)=C(t)/N and m(t)=M(t)/N. To derive kinetic equations for the time evolution of c(t) and m(t) it is noteworthy that while the conversion from C to 5mC is direct, the conversion from 5mC back to C involves a large number of intermediary steps (Figure 2A) which, as a result of the binding and unbinding kinetics, are nonlinear (Langmuir kinetics). Each of these steps requires the recruitment of different enzymes to the DNA, as well as their binding and unbinding, and eventually excision and repair of the DNA. It is clear that the conversion from 5mC to C cannot be instantaneous. Specifically, it has been shown that a large number of nonlinear reactions can be effectively described by a time delay between the first and the last reaction, Δt. This is independent of the precise form of the nonlinearities arising from these reactions. In this case, this corresponds to a time delay, Δt, between the removal of the methylation mark and the establishment of an unmodified cytosine.

Then, as the number of cytosines in any state is conserved, the structure of the differential equations describing the time evolution of c(t) and m(t) takes the symmetric form,

$$\begin{array}{c} \dot{c}\left(t\right)=k_{mc}\left[c\left(t\right),m\left(t-\Delta t\right)\right]-k_{cm}\left[c\left(t\right),m\left(t\right)\right],\\ \dot{m}\left(t\right)=k_{cm}\left[c\left(t\right),m\left(t\right)\right]-k_{mc}\left[m\left(t\right),c\left(t\right)\right], \end{array}$$

where $k_{mc}\left[c,m\right]$ and $k_{cm}\left[c,m\right]$ are the concentration-dependent rates of conversion from 5mC to C and C to 5mC, respectively. Describing the time evolution in terms of rate equations, implicitly makes the important assumption that changes in DNA methylation globally effect the genomic region under consideration. This assumption will be discussed in the following section. For the beginning of the mathematical analysis, for simplicity, it was assumed that enzyme binding to the DNA is not limited by the concentration of these enzymes, such that conversion rates are linear functions of the concentrations c(t) and m(t). The inclusion of higher order nonlinearities arising from enzyme binding and unbinding kinetics does not qualitatively alter the results, as will be shown further below.

In this simplified case, i.e. neglecting higher order nonlinearities arising from the Langmuir kinetics, the rate of loss of Cs, $k_{cm}$, is simply proportional to the concentration of cytosines that are available for conversion, c(t). Additionally, it is assumed that 5mC is autocatalytic, i.e. establishment of 5mC catalyses further methylation. There are several reports that suggest that *de novo* methylation is indeed autocatalytic: DNMT3A and DNMT3L enzymes have been shown to act cooperatively by forming heteromeric polymers and *in vitro* studies suggest that these enzymes are capable of polymerising on the DNA, thereby effectively methylating groups of adjacent cytosines at the same time (Jia et al., 2007). Further, DNMT3A/B enzymes have been shown to have increased binding affinity to 5mC and in somatic cells this leads to inhibition of DNMT3A/B degradation and an overall higher abundance of DNMT3A/B (Sharma et al., 2011). Taken together, this suggests that 5mCs catalyse *de novo* methylation of cytosines. In the simplest case, the conversion rate from C to 5mC therefore takes the form $k_{cm}\left[c\left(t\right),m\left(t\right)\right]\propto c\left(t\right)m\left(t\right)$ such that, for low methylation levels, *de novo* methylation is limited by the concentration of 5mCs while, for high methylation levels, it is limited by the concentration of Cs. Similarly, the rate of production of Cs is proportional to the number of 5mCs, and the rate of conversion is simply $k_{mc}\left[c\left(t\right),m\left(t\right)\right]\propto m\left(t\right)$, independent of c(t). DNA methylation turnover in a large genomic region is therefore effectively described by a simple set of time-delayed differential equations capturing essential properties of methylation turnover (Figure 2A),

$$\begin{array}{c} \dot{c}\left(t\right)=k_{m}m\left(t-\Delta t\right)-k_{c}c\left(t\right)m\left(t\right),\\ \dot{m}\left(t\right)=k_{c}c\left(t\right)m\left(t\right)-k_{m}m\left(t\right), \end{array}$$

where $k_{m}$ and $k_{c}$ define the effective constant rates of conversions from 5mCs and Cs, respectively.

To show that DNA methylation turnover can indeed lead to oscillatory dynamics and to investigate the conditions under which oscillations occur the differential equations were first non-dimensionalized by rescaling time such that $\tau \equiv k_{m}t$ and $\Delta \tau \equiv k_{m}\Delta t$, and concentrations such that $u\equiv \left(k_{c}/k_{m}\right)c$ and $v\equiv \left(k_{c}/k_{m}\right)m$,

$$\begin{array}{c} \dot{u}\left(\tau \right)=v\left(\tau -\Delta \tau \right)-u\left(\tau \right)v\left(\tau \right),\\ \dot{v}\left(\tau \right)=u\left(\tau \right)v\left(\tau \right)-v\left(\tau \right). \end{array}$$

This results in a system of time-delayed differential equations with a single parameter, Δτ, which qualitatively determines the dynamics. To fully define the model, initial conditions of the form $u\left(\tau \right)\equiv u_{0}\left(\tau \right)$ and $v\left(\tau \right)\equiv v_{0}\left(\tau \right)$ for $\tau^{\prime }\leq \tau \leq 0$ were supplied, effectively resulting an infinite dimensional problem. For simplicity, constant initial conditions, $u\left(\tau \right)\equiv u_{0}$ and $v\left(\tau \right)\equiv v_{0}$ for $\tau^{\prime }\leq \tau \leq 0$, were considered here. The above equations admit stationary solutions where u(t)=1 or v(t)=0, corresponding to a balance between methylation and demethylation, or a completely unmethylated genome, respectively.

Studies of time-delayed differential equations have mostly focussed on linear systems, while nonlinear systems are still poorly understood. Leaving a rigorous analytical study to future work, the dynamics of the time-delayed systems was studied using numerical integration. To obtain the period of oscillations time points where the derivative of v(τ) vanishes were determined. The period was then calculated as the average difference between next nearest roots of the derivative. Similarly, the amplitudes of u(τ) and v(τ) were defined as the average difference between two consecutive roots. Averages were taken over the last 10 periods of an overall simulation time of 1000.

Figure 2B shows numerically obtained phase portraits (${\text{u}}_{0}=1.6$, ${\text{v}}_{0}=0.4$) of the dynamics for a given set of initial conditions and varying values of the dimensionless time delay, Δτ. For small values of Δτ, the dynamics spiral inward and asymptotically focus on a singular point in phase space. With increasing values of the time delay, trajectories converge toward periodic orbits with a fixed amplitude. This behavior is characteristic of systems undergoing a *Hopf bifurcation*, where a stable fixed point of the dynamics gives rise to limit cycle behavior with increasing order parameter. While a complete characterisation of the bifurcation is beyond the scope of this work, the bifurcation was studied more rigorously by defining an order parameter as the amplitude of the oscillatory variable v(τ). With this definition, Figure 2C illustrates the abrupt onset of oscillations at a specific threshold value, $\Delta \tau^{*}$. Not unexpectedly, the period of oscillations increases linearly with the time delay and is weakly dependent on the initial conditions (Figure S3A). Hence, translating back to dimensional parameters, the period increases with the time delay, Δt, and the inverse conversion rates $1/k_{m}$ and $1/k_{c}$. Finally, to systematically study the dependence on the initial conditions the threshold delay, $\Delta \tau^{*}$, was calculated as a function of $u_{0}$ and $v_{0}$ (Figures S3B and S3C). While oscillations can occur for a broad range of initial conditions, they are most easily obtained if $v_{0}$ is broadly of order unity, i.e. the initial concentration of 5mC is of the order $k_{c}/k_{m}$. Hence, to obtain oscillatory dynamics, the net rates of methylation and de-methylation should be of the same order of magnitude.

For this simple model, oscillation occurs if the delay between the removal of the 5mC mark and the establishment of an unmodified cytosine is larger than the typical time scale of the removal of 5mC marks. Such conditions may arise if the time delay is mainly a consequence of the later steps in demethylation, such as the BER pathway. Below the threshold value, oscillations are damped on a time scale given by Δτ. Importantly, on the time scales relevant in a dynamically changing system such as primed ESCs, DNA methylation oscillations are expected even if $\Delta \tau <\Delta \tau^{*}$. Although numerical results show typical threshold values of $\Delta \tau^{*}\approx 5$, one should be careful in assigning a direct biological correspondence to this number. As is shown, the threshold value can be significantly smaller or larger if stronger nonlinearities, for example arising from enzyme binding and unbinding kinetics, are taken into account. However, as the dimensionless quantity $\Delta \tau^{*}$ is roughly of order unity, the threshold delay time is on a similar scale as other characteristic time scales in DNA methylation turnover. This indicates that oscillatory dynamics can occur under biologically plausible conditions. In addition, the emergence of limit cycle behavior straight forwardly extends to transient situations, where the concentrations of DNMT3A/B and TET1/2 enzymes are time dependent. Figure S3D shows exemplary trajectories for such a scenario.

Finally, oscillatory dynamics in C and 5mC implies temporally increased concentrations of 5hmC and other transient marks between C and 5mC. The total concentrations of states in any of the intermediary stages between 5mC and C should therefore be of the same order as the changes in concentrations in the subset of CpGs taking part in the oscillations. Therefore, increased concentrations of these states are expected in strongly oscillating regions of the genome. While estimates for the concentrations of intermediary states vary widely, it has indeed been found that 5hmC is enriched in enhancer regions for which we found the strongest oscillations (Booth et al., 2012, Feldmann et al., 2013a, Hon et al., 2014). Notably, oscillatory DNA methylation does not contradict low levels of 5caC, 5fC and 5hmC in other contexts because we observe a smaller fraction of dynamically changing CpGs there.

##### Stochastic Simulation

To illustrate how oscillatory methylation dynamics give rise qualitatively to distributions such as in Figure 2D, the random nature of the conversions between different cytosine modifications was then taken into account. The distribution observed in scBS-seq datasets arise from multiple sources of noise, most notably technical noise due to the relative low coverage of the single cell technique. The first source of noise are fluctuations arising from the stochastic conversion between C and 5mC. The probability distribution of the number of Cs and 5mCs in a given region of DNA, P(C,M,t), is governed by a set of differential equations of the form

$$\begin{array}{c} \partial_{t}P_{C,M}\left(t\right)=\alpha \left[\left(C+1\right)\left(M-1\right)P_{C+1,M-1}\left(t\right)-CMP_{C,M}\left(t\right)\right]\\ +\beta \sum_{M^{\text{′}},C^{\text{′}}=0}^{\infty }M^{\text{′}P_{M^{\text{′}},C^{\text{′}}}}\left(t-\Delta t\right)\left[P_{M+1,C-1}\left(t\right)-P_{M,C}\left(t\right)\right]. \end{array}$$

It is assumed that events at t and t−Δt are effectively decoupled. These *time-delayed Master equations* were solved by making use of an adaptation of Gillespie’s direct method for time delayed stochastic systems. Due to the limited coverage of the scBS-seq method, technical noise is a further important factor contributing to the distribution of methylation rates. With r≡M/(M+C) denoting the “true” methylation rate and s the number of informative CpGs, we modeled the number of positive reads, k, as statistically independent and following a binomial distribution,

$$R_{k|r\left(t\right),s}\left(t\right)=\frac{s!}{k!\left(s-k\right)!}r{\left(t\right)}^{k}{\left(1-r\left(t\right)\right)}^{s-k}.$$

Then, with the coverage distribution Q(s) giving the probability that a given region has s informative CpGs, the number of positive reads is distributed according to

$$R_{k}\left(t\right)=\sum_{C,M=0}^{\infty }P_{C,M}\left(t\right)\sum_{s=5}^{\infty }Q\left(s\right)\frac{s!}{k!\left(s-k\right)!}{\left(\frac{C}{C+M}\right)}^{k}{\left(1-\frac{C}{C+M}\right)}^{s-k},$$

where the sum over s starts from 5 as we removed all elements (e.g. H3K4me1 sites) with a coverage less then 5 from the statistical analysis of the scBS-seq data. Empirically, for H3K4me1 sites Q(s) can be approximated by an exponential distribution with mean 14.3. For the Supplemental Movie and Figure 2D of the main manuscript parameters were chosen as α=0.001, β=1, Δt=6. The plots and the video show the distributions of the fraction of positive reads, k/s, and they were obtained by sampling over 48,000 simulations. The resulting distributions of methylation levels recapitulate the ones observed in the single-cell experiment (Smallwood et al., 2014) (Figure 2D). As sequencing is a static measurement, it cannot be ruled out, however, that similar distributions can result from different mechanisms. This potential ambiguity necessitates the development of dynamic measurements of DNA methylation, as implemented in the 2i release experiment.

##### Structural Stability

DNA methylation turnover is a conservative process, i.e. if the cell is not in S-phase, the number of cytosines in any form remains constant over time. In conservative systems, periodic solutions are structurally unstable, such that small alterations of the dynamic rules give rise to qualitatively different dynamics. In particular, conservative systems do not give rise to limit cycle behavior. One might therefore argue that the limit cycle behavior predicted with the model might not reflect the true dynamics of methylation turnover. Indeed, by restricting the model on the C and 5mC states, the overall numbers of cytosines is not explicitly fixed. However, there are several reasons why limit cycles nevertheless can appropriately describe oscillations in DNA methylation:

1. As will be discussed below, DNA methylation turnover might be subject to global feedback by fluctuations in the concentrations of DNMT3A/B and TET1/2 enzymes. This positive feedback effectively stabilizes oscillations with respect to amplitude perturbations, thereby giving rise to stable periodic orbits.
2. Oscillations are also coupled locally by cooperative DNMT3A/B/L binding. It has been shown that local coupling can give rise to limit cycles in cyclic conservative systems.
3. Additive noise can drive conservative oscillatory systems out of states, which would otherwise arrest the dynamics, thereby effectively stabilizing oscillations.

The limit cycle oscillations emerging from our simple model are structurally robust, i.e. changes to the model should not give rise to qualitatively different behavior. In the following several structural perturbations to our model will be qualitatively investigated showing that limit cycles still occur under these conditions.

First, the model predicts vanishing *de novo* methylation in the case of completely unmethylated DNA, i.e. v=0 is a fixed point of the dynamics. This fixed point is likely to be an artefact of the simplified modeling scheme. Adding a term proportional to u(τ) to the conversion rate from C to 5mC escribes effect of *de novo* methylation which is independent of pre-existing methylation patterns. The modified system of delay differential equations reads

$$\begin{array}{c} \dot{u}\left(\tau \right)=v\left(\tau -\Delta \tau \right)-u\left(\tau \right)\left[v\left(\tau \right)+\epsilon \right],\\ \dot{v}\left(\tau \right)=u\left(\tau \right)\left[v\left(\tau \right)+\epsilon \right]-v\left(\tau \right). \end{array}$$

The dimensionless parameter ϵ denotes the relative strength of linear *de novo* methylation as compared to autocatalytic *de novo* methylation. Figure S3E shows that the transition to limit cycle behavior remains qualitatively the same, although the threshold increases with increasing values of ϵ.

Second, stronger nonlinearities were taken into account. Such nonlinearities arise, for example, due to DNA binding and unbinding kinetics of enzymes and are enhanced by the cooperative action of enzymes. In the case of DNA methylation turnover, strong nonlinearities due to the cooperative action of DNMT3A/B/L and the large number of processes involved in demethylation can indeed be expected. Therefore, systems of the following form were investigated:

$$\begin{array}{c} \dot{u}=\frac{v{\left(\tau -\Delta \tau \right)}^{n}}{A^{n}+v{\left(\tau -\Delta \tau \right)}^{n}}-\frac{{\left[u\left(\tau \right)v\left(\tau \right)\right]}^{m}}{B^{m}+{\left[u\left(\tau \right)v\left(\tau \right)\right]}^{m}},\\ \dot{v}=\frac{{\left[u\left(\tau \right)v\left(\tau \right)\right]}^{m}}{B^{m}+{\left[u\left(\tau \right)v\left(\tau \right)\right]}^{m}}-\frac{v{\left(\tau \right)}^{n}}{A^{n}+v{\left(\tau \right)}^{n}}. \end{array}$$

A and B are dimensionless quantities corresponding to threshold concentrations and the Hill coefficients m and n determine the strength of the nonlinearity of the conversion process. For example, they can be thought of the number of polymerisation processes enzymes undergo before catalysing a reaction. Depending on the parameters defining these nonlinearities, the conditions for robust oscillation may be significantly enhanced, as shown in Figure S3F, or diminished.

##### Emergence of Global Coherence in DNA Methylation Oscillations

The experimental observation of genome-wide coherence in DNA methylation (Figure 1C) implies the emergence of collective degrees of freedom. The following calculations show how, despite the complexities affecting DNA methylation on the local level, collective behavior can arise through coupling of local DNA methylation dynamics.

A single CpG can be in one of n different chemical states, such as an unmodified cytosine, bound to DNMT3A/B enzyme, a methylated cytosine, and a number of states involved in the removal of the methylation mark. As the biochemistry of DNA methylation is cyclic a phase representation for the chemical state of a CpG was chosen, where the discrete phase variable ϕ can take one out of n different values from the interval [0,2π). Following the biochemistry of DNA methylation turnover, the phase is advanced by increments of 2π/n with a rate ω, which for simplicity we take to be constant throughout the cycle. If the biochemical steps of the DNA methylation cycle are statistically independent of each other, the time evolution of the probability of finding the CpG in state ϕ follows a Master equation of the form

$$\partial_{t}\;P\left(\phi ,t\right)=\frac{n\omega }{2\pi }\left[P\left(\phi -1,t\right)-P\left(\phi ,t\right)\right].$$

The solution for delta-distributed initial conditions is simply a Poisson distribution with mean and variance ωtn/(2π). In the presence of both DNMT3s and TETs, a single cytosine is therefore, trivially, a stochastic oscillator.

However, an independent set of such stochastic oscillators, even if initiated in phase, would quickly desynchronise and cannot explain the coherent nature of DNA methylation oscillations. Single-cell bisulfite sequencing experiments in serum conditions and *in vivo* suggest that DNA methylation dynamics is correlated on the genome scale (Figures 1 and 5). To understand the emergence of coherent dynamics it is important to note that the stochastic dynamics of CpGs is weakly coupled, both globally and locally. One mechanism of positive feedback arises from DNMT3A/B binding to the DNA. It has been shown that DNMT3A/B have increased binding affinity to highly methylated DNA. In somatic cells, unbound DNMT3A/B is selectively degraded by the proteosomal pathway, such that high levels of DNA methylation effectively lead to increased concentrations of DNMT3A/B in the nucleus, while globally hypomethylated DNA reduces DNMT3A/B levels. It is likely that unbound DNMT3A/B is also selectively degraded in the much more dynamic ESCs. The global positive feedback by DNMT3A/B enzyme concentrations provides a potential mechanism for synchronising oscillations in DNA methylation. Mathematically, this feedback was taken to affect local DNA methylation with a locus specific strength $\sigma_{i}$. Reversely, the effect of local DNA methylation on the global concentration of DNMT3A/B is denoted by $\kappa_{i}$.

Further, DNMT3A/B/L molecules may form heteromers and simultaneously methylate multiple cytosines on the DNA. Beyond that, *in vitro* studies suggest that DNMT3A/B/L can polymerise on the DNA such that these interactions might even affect CpGs in low-density regions (Emperle et al., 2014). To investigate the extent to which the combination of these couplings may lead to oscillatory dynamics on the genome scale the stochastic local dynamics was combined with mathematical representations of both short-range and global interactions. To model these short-range interactions, an interaction kernel $l\left(\left|r_{i}-r_{j}\right|\right)$ was defined giving the strength of coupling between CpGs at positions $r_{i}$ and $r_{j}$. We assume these interactions to be local, i.e. $l\left(|r_{i}-r_{j}\right)$ decays much on a much shorter scale than the size of the genome. In other words, it is assumed that CpG rich regions such as CpG islands are localised. This allows the definition of a normalized kernel $K_{ij}=\lambda^{-1}l\left(\left|r_{i}-r_{j}\right|\right)$ with $\lambda =\sum_{ij}l\left(\left|r_{i}-r_{j}\right|\right)$.

Taken together, the time evolution of the distribution $P\left(\left\{\phi_{i}\right\},t\right)$ for a given configuration $\left\{\phi_{i}\right\}$ and time t is governed by Master equations of the form

$$\partial_{t}\;P\left(\left\{\phi_{i}\right\},t\right)=\sum_{i}\frac{n_{i}}{2\pi }\left[{\hat{\omega }}_{i}+\lambda \sum_{j}K_{ij}\;{\hat{\Lambda }}_{ij}\left(\phi_{j}-\phi_{i}\right)+\kappa_{i}\sum_{j\neq i}\sigma_{j}\;{\hat{\Lambda }}_{ij}\left(\phi_{j}-\phi_{i}\right)\right]P\left(\phi_{i},t\right),$$

with ${\hat{\omega }}_{i}=\left|\omega_{i}\right|\left({\hat{\phi }}_{i}^{-\text{sign}\;\omega_{i}}\right)$ and shifting operators defined as

$${\hat{\phi }}_{i}^{\pm }P\left(\phi_{1},\ldots ,\phi_{i},\ldots \phi_{N}\right)=P\left(\phi_{1},\ldots ,\phi_{i}\pm 1,\ldots ,\phi_{N},t\right).$$

The local and global coupling functions take the form

$${\hat{\Lambda }}_{ij}\left(\phi \right)=\frac{1+sin\left(\varepsilon \left[\phi +1\right]\right)}{2}{\hat{\phi }}^{-}+\frac{1-sin\left(\varepsilon \left[\phi -1\right]\right)}{2}{\hat{\phi }}^{+}-1.$$

Therefore, although the stochastic dynamics of single CpGs was initially assumed to be unidirectional, the coupling between sites induced biased diffusive dynamics. Therefore, the simplifying assumption of strict local unidirectionality does not restrict the generality of our results for the global phase dynamics as derived below. A simpler version of these equations has been studied previously in (Jörg, 2017).

To infer the collective dynamics of the stochastic phase variables we now successively coarse grain the Master equations. Specifically, at each renormalisation step, we phase average two neighbouring CpGs or blocks of CpGs with the strongest mutual coupling, $K_{i,i+1}$, and obtain a new phase variable of the form

$${\tilde{\phi }}_{i}=\frac{n_{i}\phi_{i}+n_{i+1}\phi_{i+1}}{n_{i}+n_{i+1}},$$

with $n_{i}$ being the number of CpGs in block i. We then renormalise the couplings and frequencies in order to bring the equations back into the original form. The renormalisation procedure is depicted in Figure S3G. Renormalising the dynamics in this way is typically referred to as *strong disorder renormalisation*. The implicit assumption we make in this procedure is that at high CpG densities, the effect of local coupling on the phase dynamics is stronger than the effect of global coupling and the intrinsic stochastic dynamics of CpGs. Upon renormalization, the different terms in the Master equation behave in the following ways:

The number of states in a coarse-grained phase variable increases exponentially as ${\tilde{n}}_{i}=n_{i}n_{i+1}-I/2$, where I is the number of elements in the intersection between the states $\phi_{i}$ and $\phi_{i+1}$. Upon renormalization, the phase variable quickly becomes continuous.

The new stochastic, coarse-grained phase variable ${\tilde{\phi }}_{i}$ has an effective frequency of

$${\tilde{\omega }}_{i}=\frac{n_{i}\omega_{i}+n_{i+1}\omega_{i+1}}{n_{i}+n_{i+1}}.$$

After each coarse graining, the largest element of $K_{ij}$ is removed. We renormalise K and obtain a new coupling constant

$$\tilde{\lambda }=\frac{\lambda }{n_{i}+n_{i+1}},$$

such that, for the local interactions, the Master equations read

$$\partial_{t}P\left(\left\{{\tilde{\phi }}_{i}\right\},t\right)=\sum_{i}\frac{{\tilde{n}}_{i}}{2\pi }\left[\hat{\tilde{\omega_{i}}}+\tilde{\lambda }\sum_{ij}K_{ij}\hat{\Lambda }\left({\tilde{\phi }}_{j}-{\tilde{\phi }}_{i}+\delta \right)+\ldots \right]P\left(\left\{{\tilde{\phi }}_{i}\right\},t\right),$$

where δ is to highest order a constant contribution stemming from the frequency differences between neighboring blocks of oscillators. This time-independent phase shift can be absorbed into the definition of the block phase ${\tilde{\phi }}_{i}$.

The effect of coarse graining on the global interaction terms is two-fold: The effect of the mean field on a block of phase variables scales inversely with the number of CpGs in a block, ${\tilde{\kappa }}_{i}=\kappa_{i}/n_{i}$, while the influence on the mean field scales like ${\tilde{\sigma }}_{i}=n_{i}\sigma_{i}$. Taken together, the global coupling terms remain invariant under renormalisation,

$$\partial_{t}P\left(\left\{{\tilde{\phi }}_{i}\right\},t\right)=\sum_{i}\frac{{\tilde{n}}_{i}}{2\pi }\left[\ldots +{\tilde{\kappa }}_{i}\sum_{j\neq i}{\tilde{\sigma }}_{j}\hat{\Lambda }\left({\tilde{\phi }}_{j}-{\tilde{\phi }}_{i}\right)\right]P\left(\left\{{\tilde{\phi }}_{i}\right\},t\right).$$

The equations were then successively coarse grained and renormalized until the microscopic length scale defined by the minimal distance between neighboring blocks is much larger than the local interaction range, such that $\tilde{\lambda }\leq {\tilde{\omega }}_{i},\;{\tilde{\kappa }}_{i}$. Then, the local coupling terms become irrelevant compared to the global coupling, and the dynamics on large scales is effectively described by

$$\partial_{t}P\left(\left\{{\tilde{\phi }}_{i}\right\},t\right)=\sum_{i}\frac{{\tilde{n}}_{i}}{2\pi }\left\{\hat{\tilde{\omega_{i}}}+\sum_{j\neq i}{\tilde{\kappa }}_{i}\hat{\Lambda }\left({\tilde{\phi }}_{j}-{\tilde{\phi }}_{i}\right)\right\}P\left(\left\{{\tilde{\phi }}_{i}\right\},t\right).$$

A linear noise approximation was then employed by formally writing

$$\frac{2\pi }{n_{i}}{\tilde{\phi }}_{i}\left(t\right)=\Theta_{i}\left(t\right)+\sqrt{2\pi /{\tilde{n}}_{i}}\;\xi_{i}\left(t\right)+O\left({\tilde{n}}_{i}^{-1}\right),$$

with the deterministic part following

$$\partial_{t}\Theta_{i}={\tilde{\omega }}_{i}+{\tilde{\kappa }}_{i}\sum_{j}{\tilde{\sigma }}_{i}\;sin\left(\Theta_{j}-\Theta_{i}\right)$$

and fluctuations described by

$$\frac{d}{dt}\xi_{i}=\sum_{j}{\tilde{\kappa }}_{j}cos\left(\Theta_{j}-\Theta_{i}\right)\left(\xi_{j}-\xi_{i}\right)+\sqrt{\left|{\tilde{\omega }}_{i}\right|+\sum_{j}\kappa_{j}}\;\eta_{i}\left(t\right),$$

where $\eta_{i}\left(t\right)$ is Gaussian white noise with $\langle \eta_{i}\left(t\right)\rangle =0$ and $\langle \eta_{i}\left(t\right)\eta_{j}\left(t^{\prime }\right)\rangle =\delta_{ij}\delta \left(t-t^{\prime }\right)$. By successively merging neighboring CpGs, the typical number of states, ${\tilde{n}}_{i}$, grows exponentially. Taking the continuum limit, ${\tilde{n}}_{i}\to \infty$, the discrete phase is well-approximated by ${\tilde{\phi }}_{i}\left(t\right)\to \Theta_{i}\left(t\right)$. Therefore, on large scales, the dynamics is effectively described by equations of the form

$$\partial_{t}\Theta_{i}={\tilde{\omega }}_{i}+{\tilde{\kappa }}_{i}\sum_{j}{\tilde{\sigma }}_{j}sin\left(\Theta_{j}-\Theta_{i}\right).$$

This is the heterogeneous Kuramoto model, which has been studied in the literature(Paissan and Zanette, 2008). The main results are briefly recapitulated and discussed here in the context of collective DNA methylation oscillations. In the spirit of Kuramoto’s original theory these equations can be reformulated in terms of a global phase Φ(t) and amplitude r(t) defined as the modulus and amplitude of the complex number $z\left(t\right)\equiv r\left(t\right)\text{exp}\left[i\Psi \left(t\right)\right]=N^{-1}\sum_{i}\sigma_{i}\text{exp}\left[i\phi_{i}\left(t\right)\right]$, respectively,

$$\partial_{t}\phi_{i}={\tilde{\omega }}_{i}+{\tilde{\kappa }}_{i}rsin\left(\Phi -\phi_{i}\right).$$

The condition for synchronisation, and the global amplitude r(t), can then be obtained by self-consistency. It is found that the probability of asymptotically synchronised oscillators is proportional to the coupling to the global field,

$$p_{s}\left(\delta ,\tilde{\kappa },\tilde{\sigma }\right)=\tilde{\kappa }rG\left(\Omega +\tilde{\kappa }r\;\sin\;\delta ,\tilde{\kappa },\tilde{\sigma }\right)\cos\;\delta ,$$

where δ is the phase shift between the local oscillator and the global phase and $G\left(\tilde{\omega },\tilde{\kappa },\;\tilde{\sigma }\right)$ is the probability distribution of oscillators with frequency $\tilde{\omega }$ and coupling $\tilde{\kappa }$ and $\tilde{\sigma }$. Global synchronisation occurs if

$$\int_{0}^{\infty }\tilde{\kappa }\int_{0}^{\infty }\tilde{\sigma }G\left({\tilde{\omega }}_{0},\tilde{\kappa },\tilde{\sigma }\right)d\tilde{\kappa }\;d\tilde{\sigma }\geq \frac{2}{\pi },$$

with the most abundant frequency denoted by ${\tilde{\omega }}_{0}$. More specifically, if the contribution to the mean field and its effect on the local oscillator are correlated through a relation $\tilde{\sigma }=\tilde{\sigma }\left(\tilde{\kappa }\right)$ and the intrinsic frequencies are statistically independent of the coupling, then $G\left(\tilde{\omega },\tilde{\kappa },\tilde{\sigma }\right)=g\left(\tilde{\omega }\right)h\left(\tilde{\kappa }\right)f\left(\tilde{\sigma }\right)$ and the condition for global synchronisation reduces to

$$\int_{0}^{\infty }\tilde{\kappa }h\left(\tilde{\kappa }\right)\tilde{\sigma }\left(\tilde{\kappa }\right)d\tilde{\kappa }\geq \frac{2}{\pi g\left({\tilde{\omega }}_{0}\right)},$$

In the case of DNA methylation turnover, the coupling to the mean field and the contribution of each oscillator to it are both mediated through DNMT3A/B binding, such that $\tilde{\kappa }$ and $\tilde{\sigma }$ are proportional, $\tilde{\sigma }\left(\tilde{\kappa }\right)=a\tilde{\kappa }$. Therefore, globally synchronised oscillations occur if

$$\int_{0}^{\infty }{\tilde{\kappa }}^{2}h\left(\tilde{\kappa }\right)d\tilde{\kappa }=\langle {\left(\tilde{\kappa }-\langle \tilde{\kappa }\rangle \right)}^{2}\rangle +{\langle \tilde{\kappa }\rangle }^{2}\geq \frac{2}{\pi ag\left({\tilde{\omega }}_{0}\right)}.$$

Interestingly, global synchronisation of DNA methylation oscillations is not only promoted by the average global coupling, but also by the variance thereof. The denominator, $g\left({\tilde{\omega }}_{0}\right)$, suggests that the value of the critical coupling strength is determined by the number of oscillators with intrinsic frequencies close to the typical frequency, ${\tilde{\omega }}_{0}$. In the special case of a set of homogeneous oscillators this implies asymptotic coherence for any distribution of $\tilde{\kappa }$.

In the special case of homogeneous coupling, $h\left(\tilde{\kappa }\right)=\delta \left(\tilde{\kappa }-{\tilde{\kappa }}_{0}\right)$, the synchronisation condition reads

$${\tilde{\kappa }}_{0}\geq \frac{2}{\pi g\left({\tilde{\omega }}_{0}\right)}.$$

In this case, it can be shown that the asymptotic degree of synchronisation increases according to a scaling law with the square root of the distance to the critical coupling ${\tilde{\kappa }}_{c}=2/\left[\pi g\left({\tilde{\omega }}_{0}\right)\right]$,

$$r\approx \sqrt{\frac{16}{\pi {\tilde{\kappa }}_{c}^{3}}}\sqrt{\frac{\mu }{-g^{\prime \prime }\left({\tilde{\omega }}_{0}\right)}},$$

with $\mu =\left({\tilde{\kappa }}_{0}-{\tilde{\kappa }}_{c}\right)/{\tilde{\kappa }}_{c}$ and $g^{\prime \prime }\left({\tilde{\omega }}_{0}\right)$ denotes the second derivative of the distribution of intrinsic frequencies at the position of the characteristic frequency ${\tilde{\omega }}_{0}$. For example, if $\tilde{\omega }$ is normally distributed, $g^{\prime \prime }\left({\tilde{\omega }}_{0}\right)$ is proportional to the negative standard deviation. Taken together, solutions corresponding to global synchronisation go from unstable below ${\tilde{\kappa }}_{c}$ to stable above ${\tilde{\kappa }}_{c}$.

In DNA methylation turnover, a large number of oscillators have identical intrinsic frequencies, such that $g\left({\tilde{\omega }}_{0}\right)\gg 1$ and $g^{\prime \prime }\left({\tilde{\omega }}_{0}\right)\ll 1$. In this case the synchronised state is stable even for vanishing coupling $\langle \tilde{\kappa }\rangle$ and perturbations lead to an exponentially fast relaxation back into the synchronised state. Hence, synchronisation occurs on a time scale proportional to $1/{\tilde{\kappa }}_{0}$ and preferential DNMT3A/B binding provides a potential mechanism stabilising synchronised oscillations in DNA methylation. It is unlikely that non-constant conversion rates qualitatively alter these results as synchronisation phenomena have been shown to be robust against heterogeneity in phase transition rates (Kuramoto, 1991).

This analysis shows how coherent oscillations can emerge despite the heterogeneity on the local scale. Starting from the microscopic dynamics we have derived qualitatively the same genome-scale behavior as with our phenomenological model. Although the analysis focussed on DNMT3A/B as the main driver leading to genome wide synchronisation, likely, there are many more sources driving synchronisation. For example, cell cycle periodically and homogeneously converts 5caC, 5fC and potentially other states to unmodified cytosines. This can effectively be understood as a periodic driving force giving rise to synchronised oscillations. Further, parallel scM&T-seq recently revealed that expression levels of *Tet1/2* genes are anti-correlated to methylation levels of several genomic elements in their vicinity (Angermueller et al., 2016). In particular, this holds true for a non-CGI promoter of the *Tet1* gene that is differentially methylated between cells. High methylation levels in these elements may therefore result in low *Tet* expression and a globally decreased rate of demethylation. Similarly, low methylation levels give rise to high *Tet* expression and subsequently increased demethylation. Methylation of the *Tet1* promoter and other elements in the vicinity of *Tet1/2* genes therefore effectively yields positive global feedback on DNA methylation. Elements whose methylation status influences the expression of *Tet1/2* play a special role in this scenario: they act as “pacemakers” which mediate the coupling of all other oscillators. These elements can therefore be thought of as a periodic force acting on CpGs in potentially distal parts of the genome. In fact, in a fluctuating environment such as a cell, oscillators are subject to many random stimuli. It has been theoretically shown that if these sources of noise affect all oscillators, the emergence of coherent dynamics is facilitated.

##### DNA Methylation Oscillations In Vivo

The observation of DNA methylation oscillations in the highly transient situation of the 2i release experiment suggests that these oscillations might also occur during the *de novo* methylation process *in vivo*, which has been shown to parallel the transcriptional and epigenetic dynamics of the *in vitro* experiment. During the exit from pluripotency (E4.5 to E6.5 in mouse) the global level of DNA methylation, averaged over cells levels, rises monotonically in time (Figure 6A). To investigate whether this process has an oscillatory component parallel bisulphite and RNA sequencing in single cells was performed at several time points during these stages in development (E4.5, E5.5, and E6.5). Different lineages were found in our dataset at E4.5 and E6.5, and the sample size does not allow to define an ensemble of statistically equivalent cells from the ensuing subpopulations, subsequent analysis was restricted to cells taken at E5.5. The analysis of sequencing data is complicated by the fact that sequencing only provides static information, which makes unveiling dynamic processes like methylation oscillations potentially challenging. Before aiming to reconstruct the temporal dimension in the methylation data the first step in the analysis was to ask whether statistical patterns can be extracted that allow to distinguish between different hypotheses explaining methylation heterogeneity.

To begin, it needed to be determined whether differences in global DNA methylation at E5.5 might be the result early lineage decisions, which would indicate that methylation heterogeneity is the result of a process on slower time scales than expected for oscillatory DNA methylation. No significant correlation between the expression of any gene and global DNA methylation was found. In particular, analogous to the situation in serum conditions *in vitro*, global DNA methylation levels were not correlated to the expression of the *Dnmt* and *Tet* genes (Figure 5B). To investigate whether DNA methylation correlated with the spatial location of cells, a potential indicator of post-transcriptional regulation of cell fate, all genes with (unadjusted) p-values for correlation to global H3K4me1 methylation smaller than 0.01 were selected. After adjusting for multiple testing, the expression of no gene was significantly correlated to global methylation. This list of 78 genes was then compared with a list of 462 genes that are differentially expressed along the embryonic body axis at E6.5 (Scialdone et al., 2016). There was no overlap between these sets. Taken together, this suggests that variability in DNA methylation between cells is not the result of slow effects extrinsic to DNA methylation turnover.

DNA methylation heterogeneity should therefore either stem from cell-to-cell variability in the process of global *de novo* methylation between E4.5 and E6.5, oscillatory DNA methylation turnover, or a combination of both. To distinguish these possibilities in the sequencing data biophysical modeling was employed to study the shape of distribution of global DNA methylation levels across cells over time. The first step of the analysis focused on a single cell which undergoes global *de novo* methylation. As global DNA methylation levels are typically calculated as an average over thousands of local genomic elements, cell-to-cell variability in these averages resulting from the stochastic nature of biochemical reactions is negligible compared to variations in the onset and rates of *de novo* methylation. It was assumed that the average level of DNA methylation in a given set of loci follows a sigmoidal form. Measuring time in units of the inverse global rate of *de novo* methylation and methylation levels in units of their saturation levels during later stages of development, the average global methylation level in a given set of genomic regions was parametrized as

$$\bar{m}\left(\Delta \tau \right)=\frac{1}{1+exp\left(-\Delta \tau \right)}.$$

The precise form of $\bar{m}\left(t\right)$ is empirically unknown, but the following results are insensitive to its specific choice. Importantly, variability in the rates and times of onset of *de novo* methylation can, by appropriate rescaling of time, be summarized in the single dimensionless quantity Δτ. To continue we denote by $f\left(m;\bar{m}\right)$ the distribution of methylation values across cells which would be observed in steady state conditions with average methylation level $\bar{m}$. More precisely, $f\left(m;\bar{m}\right)$ is the conditional probability that a cell has global methylation m given that the average global methylation across statistically identical cells is $\bar{m}$. With this, the probability that a cell has methylation level m at a time t is

$$p\left(m,\tau \right)=\int_{0}^{\tau }f\left(m;\bar{m}\left(\Delta \tau \right)\right)g\left(\Delta \tau ;\tau \right)d\Delta \tau ,$$

where g(Δτ;τ) is the probability that at time τ the *de novo* methylation process has been active for a time Δτ.

In order to quantitatively understand cell-to-cell variability throughout the process of *de novo* methylation three possible scenarios were investigated.

In the case that *de novo* methylation is not superimposed with oscillatory dynamics, $f\left(m;\bar{m}\right)$ is unimodal. If g(Δτ,τ) is also unimodal (e.g. because a homogeneous population of cells exits the pluripotent state in a stochastic manner), then p(m,τ) is unimodal at all times. The time evolution of p(m,τ) was obtained by numerical integration and it is depicted in Figure 5C (top).

g(Δτ;τ) can be bimodal if there is early lineage segregation of epiblast precursors, such that the exit from pluripotency and the start or the rate of *de novo* methylation are sufficiently different between two subpopulations. The time evolution of p(m,τ) in such a scenario is exemplarily shown in Figure 5C (middle). The distribution of global methylation levels in this case is bimodal only at intermediary stages of the *de novo* methylation process.

Last, if DNA methylation in a steady state scenario is oscillatory, the probability of methylation levels at the extreme points of the dynamics is higher than for intermediary levels (note that this is not necessarily the case in serum conditions, where oscillations in cells differ in amplitude and midpoint due to differential expression of *Dnmt3* and *Tet* genes). In this case $f\left(m;\bar{m}\right)$ takes a bimodal form. The shape of $f\left(m;\bar{m}\right)$ depends on the molecular details of DNA methylation turnover. For simplicity, and without loss of generality, f was approximated by a Gaussian mixture distribution,

$$f\left(m;\bar{m}\right)=\frac{1}{2\sqrt{2\pi }\sigma_{2}}e^{-\frac{{\left[x-\left(m-A/2\right)\right]}^{2}}{2\sigma_{2}}}+\frac{1}{2\sqrt{2\pi }\sigma_{1}}e^{-\frac{{\left[x-\left(m+A/2\right)\right]}^{2}}{2\sigma_{1}^{2}}},$$

where A is the amplitude of the oscillation. Progress can be made by noting that in this case p(m,τ) is bimodal if, at a given time τ, the variance of the distribution of $\bar{m}$ across different cells is smaller than the amplitude of the oscillation. This distribution is given by

$$h\left(\bar{m},\tau \right)=\int_{0}^{\tau }\delta \left(\bar{m}-\bar{m}\left(\Delta \tau \right)\right)g\left(\Delta \tau ;\tau \right)d\Delta \tau .$$

For notational simplicity the same symbol $\bar{m}$ is used for the random variable and for the functional form of the dynamics of *de novo* methylation in single cells. The time evolution of $h\left(\bar{m},\tau \right)$ was then studied separately for early times and for the long time asymptotics. In the former case $\bar{m}\left(\Delta \tau \right)$ was expanded to first order such that $\bar{m}\left(\tau \right)=u{\left(\Delta \tau \right)}^{-1}+O\left[u^{-2}\right]$ with u(Δτ)=exp(−Δτ). The variance of $h\left(\bar{m},\tau \right)$ then obeys

$$Var\left(\bar{m}\right)\equiv \int_{0}^{\infty }{\left[\bar{m}-\int_{0}^{\infty }{\bar{m}}^{\text{′}}h\left({\bar{m}}^{\text{′}},\tau \right)d{\bar{m}}^{\text{′}}\right]}^{2}h\left(\bar{m},\tau \right)d\bar{m}=u{\left(\Delta \tau \right)}^{-2}e^{\sigma^{2}}\left(e^{\sigma^{2}}-1\right).$$

Therefore, the variance of $\bar{m}$ initially increases monotonically in time.

For t→∞, $\bar{m}\left(\tau \right)$ was expanded to first order as a Laurent series, such that $\bar{m}\left(\Delta \tau \right)=1-u\left(\Delta \tau \right)+O\left[u^{2}\right]$. The variance of $h\left(\bar{m},\tau \right)$ then evolves as

$$Var\left(\bar{m}\right)=u{\left(\Delta \tau \right)}^{2}e^{\sigma^{2}}\left(e^{\sigma^{2}}-1\right).$$

Asymptotically, when average global DNA methylation approaches its maximum level, the variance decreases monotonically over time. In between these asymptotic regimes there must therefore be a maximum of the variance. Oscillations in global DNA methylation are therefore reflected in bimodality at early and late times of the global *de novo* methylation process. At intermediary times bimodality should only be observed if the amplitude of oscillations is of the same order as the global changes in DNA methylation that occur during the process of *de novo* methylation. Figure 5C (bottom) shows p(m,τ) for the oscillating case.

In summary, the three generic scenarios are clearly distinguishable by the occurrence of bimodality at different stages during the *de novo* methylation process. The observation of bimodality at E5.5, where global DNA methylation has reached its saturation level, is only compatible with an oscillatory modulation of DNA methylation. In agreement with the characterisation of oscillatory dynamics *in vitro*, a bimodal distribution of methylation levels between cells was particularly enriched at genomic regions with a CpG density between 2 and 3% (Figure 5G).

At a given time in development, different regions of the genome gain methylation in different degrees. While a large degree of this variation can be tracked back to CpG density we found that even for fixed values of the CpG density, some regions of the genome remain lowly methylated. A corollary of the theoretical results on the time evolution of bimodality is that at a given time during development, bimodality in the distribution of methylation levels across cells should particularly emerge at regions which remain lowly methylated and regions which have reached their maximum methylation levels. To test whether this pattern of oscillatory dynamics is reflected in the single-cell sequencing data tiled the genome was tiled into coverage-based windows and for each window the average methylation level across cells and the p-value for bimodality of methylation levels in the respective genomic region was calculated. Binning average methylation levels, the fraction of significantly bimodal elements (p<0.05) was obtained for each bin. Bimodality is indeed specific to elements that either remain lowly methylated at E5.5 or which have already reached the maximum value at this time (Figure 4F). As the former condition might overlap with elements that are inaccessible for DNMT3 binding, bimodality is more pronounced for higher average methylation levels.

### Quantification and Statistical Analysis

Published data sets used in analysis are listed in Table S1.

#### Bisulfite Sequencing

Raw sequence reads were trimmed to remove both poor quality calls and adapters using Trim Galore (v0.4.1, www.bioinformatics.babraham.ac.uk/projects/trim_galore/, Cutadapt version 1.8.1, parameters: −paired) (Martin, 2011). Trimmed reads were first aligned to the mouse genome in paired-end mode to be able to use overlapping parts of the reads only once while writing out unmapped singleton reads; in a second step remaining singleton reads were aligned in single-end mode. Alignments were carried out with Bismark v0.14.4 (Krueger and Andrews, 2011) with the following set of parameters: a) paired-end mode: −pbat; b) single-end mode for Read 1: −pbat; c) single-end mode for Read 2: defaults. Reads were then deduplicated with deduplicate_bismark selecting a random alignment for position that were covered more than once. CpG methylation calls were extracted from the deduplicated mapping output ignoring the first 6 bp of each read (corresponding to the 6N random priming oligos) using the Bismark methylation extractor (v0.14.4) with the following parameters: a) paired-end mode: −ignore 6 --ignore_r2 6; b) single-end mode: −ignore 6. SeqMonk version 0.32 was used to compute methylation rates and coverage. To QC BS-seq data, pairwise Pearson correlation coefficients were calculated using methylation levels averaged over 10kb tiles. Replicates within the same time point were on average more highly correlated than between time points (*r* = 0.885 versus 0.866). For subsequent analyses, replicates were merged.

Further statistical analysis was performed by custom scripts in Perl and R. Averages were weighted by coverage depth. For enhancers, we took into account regions which had higher total coverage depth throughout the time course than the median. For exons and promoters, which include regions with significantly varying CpG density, we filtered for regions below the 1^st^ quartile in CpG density and above the 95^th^ percentile in total coverage depth to calculate average methylation levels. To estimate the bounds of technical noise we followed standard methods by considering methylation of CpGs as statistically independent, such that the total number of methylation reads in a given region follows a binomial distribution. For each genomic region we calculated the distribution of methylation levels under the null hypothesis from 1000 binomial samples, given by the same local average coverage and methylation rate found in the experiment. Confidence bounds were then obtained from the distribution of weighted averages over all regions.

Spectral analysis is complicated by the fact that, given the temporal resolution of the time series, statistical significance in periodicity cannot be rigorously established on the level of single elements after multiple testing correction. Rather, we investigated whether parallel oscillatory dynamics is enriched over many genomic elements. To determine the distribution of spectral powers under the null hypothesis, we sampled spectral powers using the Lomb-Scargle method over 2.5e7 uncorrelated and normally distributed time series, taking into account the possibility of artificial spectral peaks that arise from the non-uniformity of time points. The average spectral power over a large set of genomic regions for a given period (or frequency) is normally distributed and we used t tests to determine statistical significance for the enrichment of periods with respect to the null hypothesis. For spectral analysis, slow trends in average DNA methylation levels were removed by considering the residuals after fitting to a second order polynomial. It is important to note that the limited number of time points and their non-uniformity gives rise to correlations in spectral powers as well as a discrete set of possible enriched periods, which is reflected in a periodicity in the average spectral powers.

To estimate the amplitude of the oscillations (excess variance) we rescaled the weighted variance of methylation levels across time points by the variance of methylation levels expected from a Bernoulli trial of a length corresponding to the average coverage $\left(\bar{c}\right)$ and average methylation level $\left(\bar{m}\right)$ for a given genomic window (technical variability), $Var\left(m\right)/\left[\bar{m}\left(1-\bar{m}\right)/\bar{c}\right]$.

#### Amplicon Bisulfite Sequencing

The first 8 bp of Read 2, representing unique molecular identifiers (UMIs), were removed and written into the sequence IDs instead. The reads were then subjected to adapter and quality trimming using Trim Galore (v0.4.2; default parameters) and aligned to the mouse genome (build GRCm38) using Bismark (v0.16.3, parameters: −non_directional). After mapping reads were deduplicated using the UMI-mode of deduplicate_bismark (options --bam --barcode).

For spectral analysis we filtered for data points with more than 100 reads and used elements with more than 20 valid time points. (n = 16 for release and n = 16 for control). Spectral analysis was performed using the Lomb-Scargle method with a scanning interval ranging from 90 to 180 minutes. Spectral peaks at the boundaries of the scanning interval were discarded.

#### Knock-out ESC and EB scBS-Seq Data Processing

Raw sequence reads had the first 6 base pairs clipped off the 5’ end to remove 6N random priming portion of the reads, and were also trimmed to remove both poor quality calls and adapters using Trim Galore (v0.4.1, www.bioinformatics.babraham.ac.uk/projects/trim_galore/, parameters: −clip_r1 6, Cutadapt v1.8.1). Remaining sequences were then aligned to the mouse genome (build GRCm38) with Bismark (v0.14.5) in single-end mode (parameters: −non_directional). Methylation calls were extracted after duplicate sequences had been excluded.

#### RNA-Seq Data Analysis

Read counts associated with different genomic features where obtained using Bioconductor’s GenomicAlignments package in R. We excluded H3K4me1 regions that overlap with exons. For the interpretation of read counts we needed to separate technical from biological variability between time points. While this potentially ambiguous we reasoned that rapid changes associated with DNA methylation oscillations must be less pronounced in transcripts with significantly longer average lifetime than the period of DNA methylation oscillations. With long-lived transcripts as a reference we focussed our analysis on short-lived transcripts, such as reads aligned to enhancer or intron regions. To this end, we calculated normalisation factors using size factor normalisation on transcripts with an average lifetime of more than the median lifetime of all transcripts (corresponding to roughly 7 hours, (Sharova et al., 2009b)). All downstream analysis was performed on log-transformed normalised read counts, after adding an offset of 1.

#### scBS-Seq Data Analysis

For violin plots shown in Figures 1 and Figures S1 and S2, SeqMonk version 0.32 was used to compute methylation rates, using annotated loci (Table S1) with more than 5 CpGs each containing at least one read. For each cell, all sites with DNA methylation values were included in beanplots prepared using R (beanwidth = 10).

To estimate biological variability of DNA methylation between cells (Figure 4C and S6A) we rescaled the weighted variance of methylation levels between cells by the variance of methylation levels expected from a Bernoulli trial of a length corresponding to the average coverage $\left(\bar{c}\right)$ and average methylation level $\left(\bar{m}\right)$ for a given genomic window (technical variability), $Var\left(m\right)/\left[\bar{m}\left(1-\bar{m}\right)/\bar{c}\right]$.

#### Embryo scM&T-Seq Analysis

Data processing was performed as previously described (Angermueller et al., 2016).

Downstream analysis was performed using custom scripts in Perl and R. Averages over methylation rates and correlations were weighted by coverage depth. For weighted correlation between *Dnmt*/*Tet* expression and global methylation we took the difference of the average log-transformed normalized read counts of the *Dnmt3a/b/l/1* genes and *Tet1/2/3* genes, respectively, after removal of dropouts. For a given cell, to estimate developmental age we tiled the genome into regions of 100 informative CpGs (more than one read each). We then filtered for tiles with CpG density between 10% and 15%. Pseudo time was defined as the average fraction of forward reads in these regions, $pt=\;N^{-1}\sum_{i}f_{i}/\left(f_{i}+b_{i}\right)$, where $f_{i}$ and $b_{i}$ are the number of forward and backwards reads in a region, respectively, and *N* Is the total number of regions under consideration. The resulting pseudo time series where then detrended using linear regression and rescaled by the expected technical variance in methylation levels.

### Data and Software Availability

The accession number for the sequencing datasets reported in this paper is GEO.

## Supporting Citations

The following references appear in Supplemental Information: Buecker et al., 2014, Factor et al., 2014, Illingworth et al., 2010, Scialdone et al., 2016.

## Supplemental Information

Document S1. Figures S1–S6 and Tables S1, S2, S4, and S5

Table S3. Details of Amplicons Studied by AmpBS-Seq Including Genomic Co-ordinates and Primer Sequences, Related to Figure 3 and STAR Methods Loci were selected based on oscillations observed in the initial 2i release experiment or on correlations between DNA methylation and gene expression in scM&T-seq data (Angermueller et al., 2016). In some cases, neighboring loci have been considered together in “pooled amplicons.” The reverse primers include an 8N unique molecular identifier for exclusion of PCR duplicates. Results of spectral analysis are also shown.

Document S2. Article plus Supplemental Information

## References

[bib1] Angermueller C., Clark S.J., Lee H.J., Macaulay I.C., Teng M.J., Hu T.X., Krueger F., Smallwood S.A., Ponting C.P., Voet T. (2016). Parallel single-cell sequencing links transcriptional and epigenetic heterogeneity. Nat. Methods.

[bib2] Atlasi Y., Stunnenberg H.G. (2017). The interplay of epigenetic marks during stem cell differentiation and development. Nat. Rev. Genet..

[bib3] Auclair G., Guibert S., Bender A., Weber M. (2014). Ontogeny of CpG island methylation and specificity of DNMT3 methyltransferases during embryonic development in the mouse. Genome Biol..

[bib4] Baubec T., Colombo D.F., Wirbelauer C., Schmidt J., Burger L., Krebs A.R., Akalin A., Schübeler D. (2015). Genomic profiling of DNA methyltransferases reveals a role for DNMT3B in genic methylation. Nature.

[bib5] Berry S., Dean C., Howard M. (2017). Slow chromatin dynamics allow Polycomb target genes to filter fluctuations in transcription factor activity. Cell Syst.

[bib6] Bintu L., Yong J., Antebi Y.E., McCue K., Kazuki Y., Uno N., Oshimura M., Elowitz M.B. (2016). Dynamics of epigenetic regulation at the single-cell level. Science.

[bib7] Booth M.J., Branco M.R., Ficz G., Oxley D., Krueger F., Reik W., Balasubramanian S. (2012). Quantitative sequencing of 5-methylcytosine and 5-hydroxymethylcytosine at single-base resolution. Science.

[bib8] Boroviak T., Loos R., Lombard P., Okahara J., Behr R., Sasaki E., Nichols J., Smith A., Bertone P. (2015). Lineage-specific profiling delineates the emergence and progression of naive pluripotency in mammalian embryogenesis. Dev. Cell.

[bib9] Boyle A.P., Davis S., Shulha H.P., Meltzer P., Margulies E.H., Weng Z., Furey T.S., Crawford G.E. (2008). High-resolution mapping and characterization of open chromatin across the genome. Cell.

[bib66] Buecker C., Srinivasan R., Wu Z., Calo E., Acampora D., Faial T., Simeone A., Tan M., Swigut T., Wysocka J. (2014). Reorganization of enhancer patterns in transition from naive to primed pluripotency. Cell Stem Cell.

[bib10] Buenrostro J.D., Giresi P.G., Zaba L.C., Chang H.Y., Greenleaf W.J. (2013). Transposition of native chromatin for fast and sensitive epigenomic profiling of open chromatin, DNA-binding proteins and nucleosome position. Nat. Methods.

[bib11] Chambers I., Silva J., Colby D., Nichols J., Nijmeijer B., Robertson M., Vrana J., Jones K., Grotewold L., Smith A. (2007). Nanog safeguards pluripotency and mediates germline development. Nature.

[bib12] Clark S.J., Smallwood S.A., Lee H.J., Krueger F., Reik W., Kelsey G. (2017). Genome-wide base-resolution mapping of DNA methylation in single cells using single-cell bisulfite sequencing (scBS-seq). Nat. Protoc..

[bib13] Creyghton M.P., Cheng A.W., Welstead G.G., Kooistra T., Carey B.W., Steine E.J., Hanna J., Lodato M.A., Frampton G.M., Sharp P.A. (2010). Histone H3K27ac separates active from poised enhancers and predicts developmental state. Proc Natl. Acad. Sci. USA.

[bib14] Du J., Johnson L.M., Jacobsen S.E., Patel D.J. (2015). DNA methylation pathways and their crosstalk with histone methylation. Nat. Rev. Mol. Cell. Biol..

[bib15] Emperle M., Rajavelu A., Reinhardt R., Jurkowska R.Z., Jeltsch A. (2014). Cooperative DNA binding and protein/DNA fiber formation increases the activity of the Dnmt3a DNA methyltransferase. J. Biol. Chem..

[bib67] Factor D.C., Corradin O., Zentner G.E., Saiakhova A., Song L., Chenoweth J.G., McKay R.D., Crawford G.E., Scacheri P.C., Tesar P.J. (2014). Epigenomic Comparison Reveals Activation of “Seed” Enhancers during Transition from Naive to Primed Pluripotency. Cell Stem Cell.

[bib16] Farlik M., Sheffield N.C., Nuzzo A., Datlinger P., Schönegger A., Klughammer J., Bock C. (2015). Single-cell DNA methylome sequencing and bioinformatic inference of epigenomic cell-state dynamics. Cell. Rep..

[bib17] Feldmann A., Ivanek R., Murr R., Gaidatzis D., Burger L., Schübeler D. (2013). Transcription factor occupancy can mediate active turnover of DNA methylation at regulatory regions. PLOS Genet..

[bib18] Ficz G., Hore T.A., Santos F., Lee H.J., Dean W., Arand J., Krueger F., Oxley D., Paul Y.L., Walter J. (2013). FGF signaling inhibition in ESCs drives rapid genome-wide demethylation to the epigenetic ground state of pluripotency. Cell Stem Cell.

[bib19] Gaidatzis D., Burger L., Murr R., Lerch A., Dessus-Babus S., Schübeler D., Stadler M.B. (2014). DNA sequence explains seemingly disordered methylation levels in partially methylated domains of Mammalian genomes. PLOS Genet..

[bib20] Habibi E., Brinkman A.B., Arand J., Kroeze L.I., Kerstens H.H., Matarese F., Lepikhov K., Gut M., Brun-Heath I., Hubner N.C. (2013). Whole-genome bisulfite sequencing of two distinct interconvertible DNA methylomes of mouse embryonic stem cells. Cell Stem Cell.

[bib21] Haerter J.O., Lövkvist C., Dodd I.B., Sneppen K. (2014). Collaboration between CpG sites is needed for stable somatic inheritance of DNA methylation states. Nucleic Acids Res..

[bib22] Hayashi K., de Sousa Lopes S.M.C., Tang F., Lao K., Surani M.A. (2008). Dynamic equilibrium and heterogeneity of mouse pluripotent stem cells with distinct functional and epigenetic states. Cell Stem Cell.

[bib23] Hiratani I., Ryba T., Itoh M., Rathjen J., Kulik M., Papp B., Fussner E., Bazett-Jones D.P., Plath K., Dalton S. (2010). Genome-wide dynamics of replication timing revealed by in vitro models of mouse embryogenesis. Genome Res..

[bib24] Hon G.C., Rajagopal N., Shen Y., McCleary D.F., Yue F., Dang M.D., Ren B. (2013). Epigenetic memory at embryonic enhancers identified in DNA methylation maps from adult mouse tissues. Nat. Genet..

[bib25] Hon G.C., Song C.X., Du T., Jin F., Selvaraj S., Lee A.Y., Yen C.A., Ye Z., Mao S.Q., Wang B.A. (2014). 5mC oxidation by Tet2 modulates enhancer activity and timing of transcriptome reprogramming during differentiation. Mol. Cell.

[bib26] Hooper M., Hardy K., Handyside A., Hunter S., Monk M. (1987). HPRT-deficient (Lesch-Nyhan) mouse embryos derived from germline colonization by cultured cells. Nature.

[bib27] Hu X., Zhang L., Mao S.Q., Li Z., Chen J., Zhang R.R., Wu H.P., Gao J., Guo F., Liu W. (2014). Tet and TDG mediate DNA demethylation essential for mesenchymal-to-epithelial transition in somatic cell reprogramming. Cell Stem Cell.

[bib68] Illingworth R.S., Gruenewald-Schneider U., Webb S., Kerr A.R., James K.D., Turner D.J., Smith C., Harrison D.J., Andrews R., Bird A.P. (2010). Orphan CpG islands identify numerous conserved promoters in the mammalian genome. PLoS Genet.

[bib28] Iurlaro M., von Meyenn F., Reik W. (2017). DNA methylation homeostasis in human and mouse development. Curr. Opin. Genet. Dev..

[bib29] Jia D., Jurkowska R.Z., Zhang X., Jeltsch A., Cheng X. (2007). Structure of Dnmt3a bound to Dnmt3L suggests a model for de novo DNA methylation. Nature.

[bib30] Jörg D.J. (2017). Stochastic Kuramoto oscillators with discrete phase states. Phys. Rev. E..

[bib31] Kalkan T., Olova N., Roode M., Mulas C., Lee H.J., Nett I., Marks H., Walker R., Stunnenberg H.G., Lilley K.S. (2017). Tracking the embryonic stem cell transition from ground state pluripotency. Development.

[bib32] Kobayashi T., Kageyama R. (2011). Hes1 oscillations contribute to heterogeneous differentiation responses in embryonic stem cells. Genes.

[bib33] Kolodziejczyk A.A., Kim J.K., Tsang J.C., Ilicic T., Henriksson J., Natarajan K.N., Tuck A.C., Gao X., Bühler M., Liu P. (2015). Single cell RNA-sequencing of pluripotent states unlocks modular transcriptional variation. Cell Stem Cell.

[bib34] Krueger F., Andrews S.R. (2011). Bismark: a flexible aligner and methylation caller for bisulfite-Seq applications. Bioinformatics.

[bib35] Kunz C., Focke F., Saito Y., Schuermann D., Lettieri T., Selfridge J., Schär P. (2009). Base excision by thymine DNA glycosylase mediates DNA-directed cytotoxicity of 5-fluorouracil. PLOS Biol..

[bib36] Kuramoto Y. (1991). Collective synchronization of pulse-coupled oscillators and excitable units. Physica D: Nonlinear Phenomena.

[bib37] Lee H.J., Hore T.A., Reik W. (2014). Reprogramming the methylome: erasing memory and creating diversity. Cell Stem Cell.

[bib38] Leitch H.G., McEwen K.R., Turp A., Encheva V., Carroll T., Grabole N., Mansfield W., Nashun B., Knezovich J.G., Smith A. (2013). Naive pluripotency is associated with global DNA hypomethylation. Nat. Struct. Mol. Biol..

[bib39] Lövkvist C., Dodd I.B., Sneppen K., Haerter J.O. (2016). DNA methylation in human epigenomes depends on local topology of CpG sites. Nucleic Acids Res.

[bib70] Macaulay I.C., Teng M.J., Haerty W., Kumar P., Ponting C.P., Voet T. (2016). Separation and parallel sequencing of the genomes and transcriptomes of single cells using G&T-seq. Nat Protocols.

[bib40] Martin M. (2011). Cutadapt removes adapter sequences from high-throughput sequencing reads. EMBnet j.

[bib41] Métivier R., Gallais R., Tiffoche C., Le Péron C., Jurkowska R.Z., Carmouche R.P., Ibberson D., Barath P., Demay F., Reid G. (2008). Cyclical DNA methylation of a transcriptionally active promoter. Nature.

[bib42] Mohammed H., Hernando-Herraez I., Savino A., Scialdone A., Macaulay I., Mulas C., Chandra T., Voet T., Dean W., Nichols J. (2017). Single-cell landscape of transcriptional heterogeneity and cell fate decisions during mouse early gastrulation. Cell Rep..

[bib43] Ooi S.K., Qiu C., Bernstein E., Li K., Jia D., Yang Z., Erdjument-Bromage H., Tempst P., Lin S.P., Allis C.D. (2007). DNMT3L connects unmethylated lysine 4 of histone H3 to de novo methylation of DNA. Nature.

[bib44] Paissan G.H., Zanette D.H. (2008). Synchronization of phase oscillators with heterogeneous coupling: a solvable case. Physica D: Nonlinear Phenomena.

[bib45] Peng G., Suo S., Chen J., Chen W., Liu C., Yu F., Wang R., Chen S., Sun N., Cui G. (2016). Spatial transcriptome for the molecular annotation of lineage fates and cell identity in mid-gastrula mouse embryo. Dev. Cell.

[bib46] Picelli S., Faridani O.R., Björklund A.K., Winberg G., Sagasser S., Sandberg R. (2014). Full-length RNA-seq from single cells using Smart-seq2. Nat. Protoc..

[bib47] Quail M.A., Otto T.D., Gu Y., Harris S.R., Skelly T.F., McQuillan J.A., Swerdlow H.P., Oyola S.O. (2011). Optimal enzymes for amplifying sequencing libraries. Nat. Methods.

[bib48] Scialdone A., Tanaka Y., Jawaid W., Moignard V., Wilson N.K., Macaulay I.C., Marioni J.C., Göttgens B. (2016). Resolving early mesoderm diversification through single-cell expression profiling. Nature.

[bib49] Seisenberger S., Andrews S., Krueger F., Arand J., Walter J., Santos F., Popp C., Thienpont B., Dean W., Reik W. (2012). The dynamics of genome-wide DNA methylation reprogramming in mouse primordial germ cells. Mol. Cell.

[bib50] Sharma S., De Carvalho D.D., Jeong S., Jones P.A., Liang G. (2011). Nucleosomes containing methylated DNA stabilize DNA methyltransferases 3A/3B and ensure faithful epigenetic inheritance. PLOS Genet..

[bib51] Sharova L.V., Sharov A.A., Nedorezov T., Piao Y., Shaik N., Ko M.S.H. (2009). Database for mRNA half-life of 19 977 genes obtained by DNA microarray analysis of pluripotent and differentiating mouse embryonic stem cells. DNA Res..

[bib52] Sharova L.V., Sharov A.A., Nedorezov T., Piao Y., Shaik N., Ko M.S.H. (2009). Database for mRNA half-life of 19 977 genes obtained by DNA microarray analysis of pluripotent and differentiating mouse embryonic stem cells. DNA Res.

[bib53] Shah S., Takei Y., Zhou W., Lubeck E., Yun J., Eng C.-H.L., Koulena N., Cronin C., Karp C., Liaw E.J. (2018). Dynamics and spatial genomics of the nascent transcriptome by intron seqFISH. Cell.

[bib54] Singer Z.S., Yong J., Tischler J., Hackett J.A., Altinok A., Surani M.A., Cai L., Elowitz M.B. (2014). Dynamic heterogeneity and DNA methylation in embryonic stem cells. Mol. Cell.

[bib55] Singh A.M., Hamazaki T., Hankowski K.E., Terada N. (2007). A heterogeneous expression pattern for Nanog in embryonic stem cells. Stem Cells.

[bib56] Smallwood S.A., Lee H.J., Angermueller C., Krueger F., Saadeh H., Peat J., Andrews S.R., Stegle O., Reik W., Kelsey G. (2014). Single-cell genome-wide bisulfite sequencing for assessing epigenetic heterogeneity. Nat Methods.

[bib57] Smith Z.D., Chan M.M., Mikkelsen T.S., Gu H., Gnirke A., Regev A., Meissner A. (2012). A unique regulatory phase of DNA methylation in the early mammalian embryo. Nature.

[bib58] Solter D., Knowles B.B. (1975). Immunosurgery of mouse blastocyst. Proc. Natl. Acad. Sci. USA.

[bib59] Stadler M.B., Murr R., Burger L., Ivanek R., Lienert F., Schöler A., van Nimwegen E., Wirbelauer C., Oakeley E.J., Gaidatzis D. (2011). DNA-binding factors shape the mouse methylome at distal regulatory regions. Nature.

[bib60] Takashima Y., Guo G., Loos R., Nichols J., Ficz G., Krueger F., Oxley D., Santos F., Clarke J., Mansfield W. (2014). Resetting transcription factor control circuitry toward ground-state pluripotency in human. Cell.

[bib61] Torres-Padilla M.E., Chambers I. (2014). Transcription factor heterogeneity in pluripotent stem cells: a stochastic advantage. Development.

[bib62] Toyooka Y., Shimosato D., Murakami K., Takahashi K., Niwa H. (2008). Identification and characterization of subpopulations in undifferentiated ES cell culture. Development.

[bib63] von Meyenn F., Iurlaro M., Habibi E., Liu N.Q., Salehzadeh-Yazdi A., Santos F., Petrini E., Milagre I., Yu M., Xie Z. (2016). Impairment of DNA methylation maintenance is the main cause of global demethylation in naive embryonic stem cells. Mol. Cell.

[bib64] Wang L., Zhang J., Duan J., Gao X., Zhu W., Lu X., Yang L., Zhang J., Li G., Ci W. (2014). Programming and inheritance of parental DNA methylomes in mammals. Cell.

[bib65] Wu H., Zhang Y. (2014). Reversing DNA methylation: mechanisms, genomics, and biological functions. Cell.

