# Supplementary material for: Genome-Scale Oscillations in DNA Methylation during Exit from Pluripotency
Source: Cell Syst. 2018 Jul 25;7(1):63–76.e12. doi: 10.1016/j.cels.2018.06.012 (PMC6066359; doi:10.1016/j.cels.2018.06.012)
Supplement: Document S1. Figures S1–S6 and Tables S1, S2, S4, and S5 [file mmc1.pdf]

Cell Systems, Volume 7

Supplemental Information

Genome-Scale Oscillations

in DNA Methylation

during Exit from Pluripotency

Steffen Rulands, Heather J. Lee, Stephen J. Clark, Christof Angermueller, Sébastien A. Smallwood, Felix Krueger, Hisham Mohammed, Wendy Dean, Jennifer Nichols, Peter Rugg-Gunn, Gavin Kelsey, Oliver Stegle, Benjamin D. Simons, and Wolf Reik

Supplemental Information

Supplemental Legends

Figures S1-6

Tables S1, S2, S4 and S5

Supplemental Legends

Figure S1. DNA methylation variance in naïve and primed ESCs. Related to Figure 1 and Table S1. **(A)** A comparison of context-specific variance in DNA methylation was determined using scBS-seq data from 12 naïve and 20 primed ESCs (Smallwood et al., 2014) at different genomic contexts. Variance was calculated for all 3kb tiles across the genome and is compared to genomic loci defined using published data (Table S1). All differences between naïve and primed conditions are statistically significant ($p<3.2e-13$, t-test). IAP: interstitial A Particle. TSS: transcription start site (promoter). **(B)** Mean DNA methylation levels in single cells in late replicating regions and early replicating regions (Hiratani et al., 2010). Error bars signify 95% confidence intervals. **(C)** Violin plots of DNA methylation in primed ESCs for various genomic contexts (Table S1). Each violin represents an individual cell. **(D)** Violin plots of DNA methylation in naïve and primed ESCs for H3K4me1 sites defined in naïve ESCs (Buecker et al., 2014). Each violin represents an individual cell.

Figure S2. DNA methylation heterogeneity depends on co-expression of *Dnmt3* and *Tet* enzymes. Related to Figure 1 and Table S2. **(A)** Violin plots of DNA methylation at primed ESC H3K4me1 sites for cells from day 1 and day 3 of embryoid body (EB) differentiation. Each violin represents an individual cell. **(B)** RT-PCR of *Dnmt3a/b* and *Tet1/2* in naïve and primed ESCs and during EB differentiation. Expression is normalised to *Atp5b*. **(C)** DNA methylation heterogeneity is specific to the 'More Pluripotent' sub-population of primed ESCs. Top left: To identify primed ESC sub-populations hierarchical clustering was performed as previously described (Kolodziejczyk et al., 2015), using 61

primed ESCs analysed by scM&T-seq (Angermueller et al., 2016), and a panel of 86 pluripotency and differentiation genes (Table S2). Top right: Expression of selected genes of interest in the 3 subgroups of primed ESCs. Bottom panels: Violin plots of DNA methylation at H3K4me1 sites for cells from the 3 subgroups of primed ESCs. Each violin represents an individual cell. DNA methylation heterogeneity is observed only in the 'More Pluripotent' population that has the highest expression of *Dnmt3* and *Tet* enzymes. **(D)** Scatter plot of log expression values of selected genes involved in DNA methylation turnover as a function of global DNA methylation in H3K4me1 regions. Each point is a cell cultured in primed conditions with size and transparency indicating global scBS-seq coverage.

Fig. S3. Mathematical analysis of the biophysical model. Related to Figure 2 and STAR Methods. **(A)** Period as a function of the delay time, and v_0 is depicted by color code ($0.1 \leq \mathrm{u}_0, \mathrm{v}_0 \leq 2$). See STAR methods for details. **(B)** Threshold value of the delay time, τ^*, as a function of the initial conditions. Blue areas signify absence of sustained oscillations. **(C)** Threshold value for fixed $\mathrm{u}_0 = 1.6$ (black dots) and interpolating line (blue). **(D)** Numerical solutions with time dependent rates $k_m(t)$and $k_c(t)$. Top: The rates were chosen to mimic the down regulation of Tet (solid line) and upregulation of Dnmt3a/b (dashed line) during the loss of pluripotency. Bottom: Concentration of methylated CpGs, m(t), over the same time interval. We find the emergence of transient oscillations during the period of co-expression of the two groups of genes. **(E)** Amplitude dependence of oscillations as a function of time-delay obtained from solving a model a linear component in de-novo methylation with $\epsilon = 0.1$. **(F)** Amplitude dependence of oscillations as a function of time-delay obtained from solving a model taking into account non-linearities arising from enzyme binding and unbinding, with $A = B = 0.5$ and $m = n = 4$. In both cases we set $u_0 = 1.6$ and $v_0 = 0.4$. The dimensionless concentrations $\mathrm{u}_0, \mathrm{v}_0$ and other mathematical details are defined in Star Methods. **(G)** Illustration of an exemplary case of the coarse graining procedure. At each step, the two strongest interacting CpGs (black) or blocks of CpGs (red) are phase-averaged. Couplings and intrinsic frequencies are then renormalised to bring the Master equations back into the original form.

Fig. S4. Oscillatory dynamics after 2i release. Related to Figure 3. **(A)** Average DNA methylation in H3K4me1 regions. Dotted lines denote different quantiles of the expected variability under the null hypothesis that the observed pattern is the result of noise (grey: 5% and 95%, blue: 32% and 68%, red: median). The oscillatory pattern exceeds what is expected by technical noise. **(B)** Average DNA methylation in regions marked by H3K27ac with the same coverage filter applied as in Figure 3A (n=6432). Shaded areas in (A) and (B) denote standard error. **(C)** Average spectral densities for different genomic features. Red dots denote significant enrichment of a given period ($p<0.05$). Thin lines denote standard error. **(D)** Methylation levels at exemplary enhancer elements as measured by whole genome BS-seq. Dots denote methylation calls and shaded regions signify standard errors.

Fig. S5. DNA methylation oscillations in Amplicon sequencing experiments. Related to Figure 4. DNA methylation at exemplary enhancer elements in **(A)** primed conditions after 2i release and **(B)** control conditions (cells maintained in naïve conditions). Each plot represents an amplicon. Time courses showing statistically significant oscillatory patterns are marked in red.

Fig. S6. DNA methylation oscillations *in vivo.* Related to Figures 5 and 6, and STAR Methods. **(A)** Biological variability (see STAR methods for mathematical definition) for different genomic features at each of the three analysed stages during development. **(B)** Scatter plot of log expression values of selected genes involved in DNA methylation turnover as a function of global DNA methylation in H3K4me1 regions. Each point is a cell taken at E5.5 with size and transparency indicating global scBS-seq coverage. **(C)** Heat maps depicting pairwise correlation coefficients between gene expression levels or global DNA methylation in H3K4me1 regions. **(D)** Adjusted average DNA methylation in regions with different ranges of CpG densities as a function of methylation pseudo time. Solid lines show local regression using the Loess method.

Table S1: Published data sets used in this study. Related to Figures 1, S1 and 2.

Table S2: Pluripotency and differentiation genes used to cluster primed ESCs in Figure S2C. Related to Figures 1 and S2. Taken from Angermueller et al., 2016.

Table S4: Primer sequences for qPCR analysis. Related to Figures 1 and S2, and STAR Methods.

Table S5: Indexing primers used for Amplicon BS-seq. Related to Figure 3 and STAR Methods. *indicates phosphorothioates between two bases where an s-linkage should occur.

Figure S1. Related to Figure 1

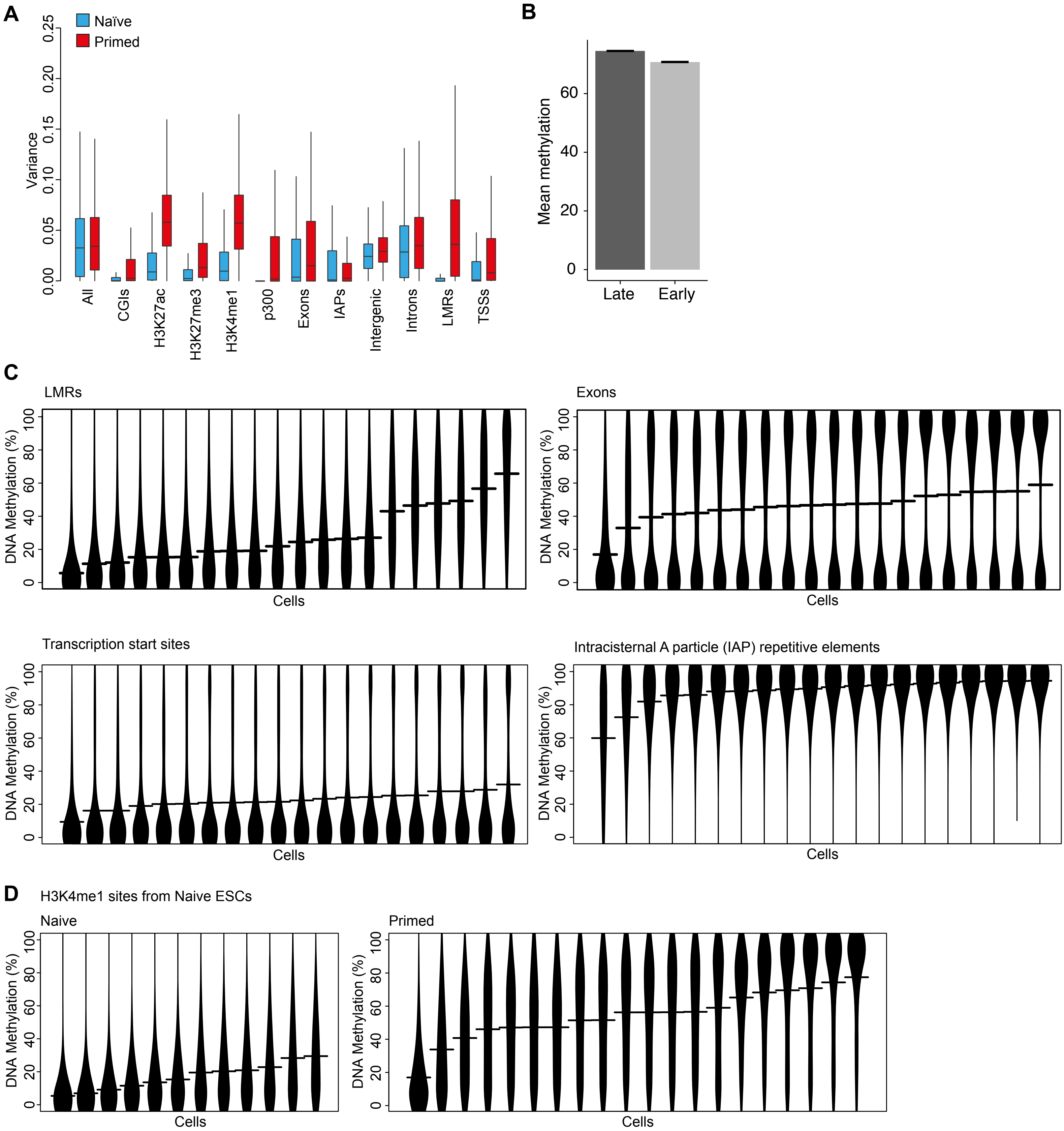

Figure S2. Related to Figure 1

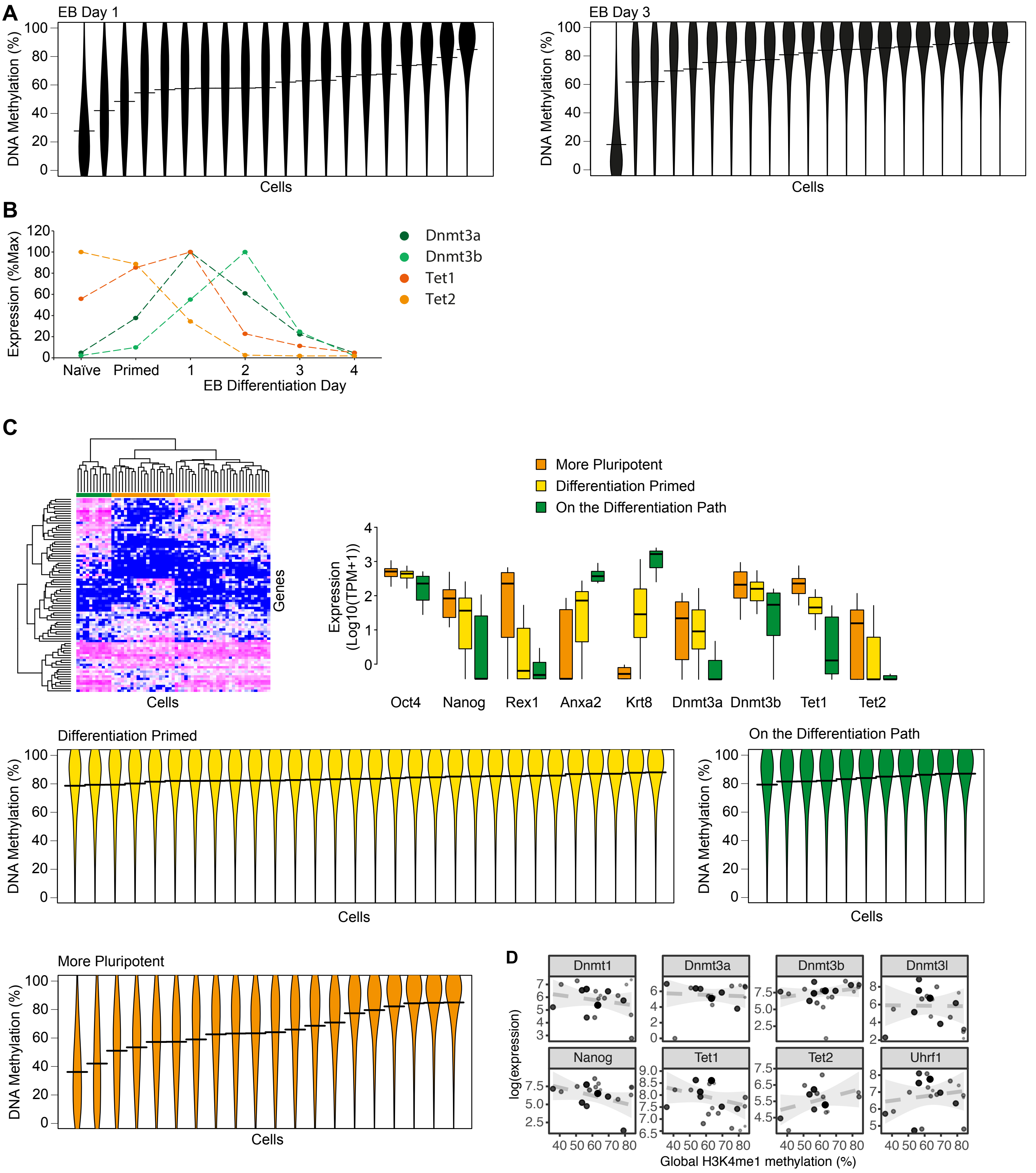

Figure S3. Related to Figure 2

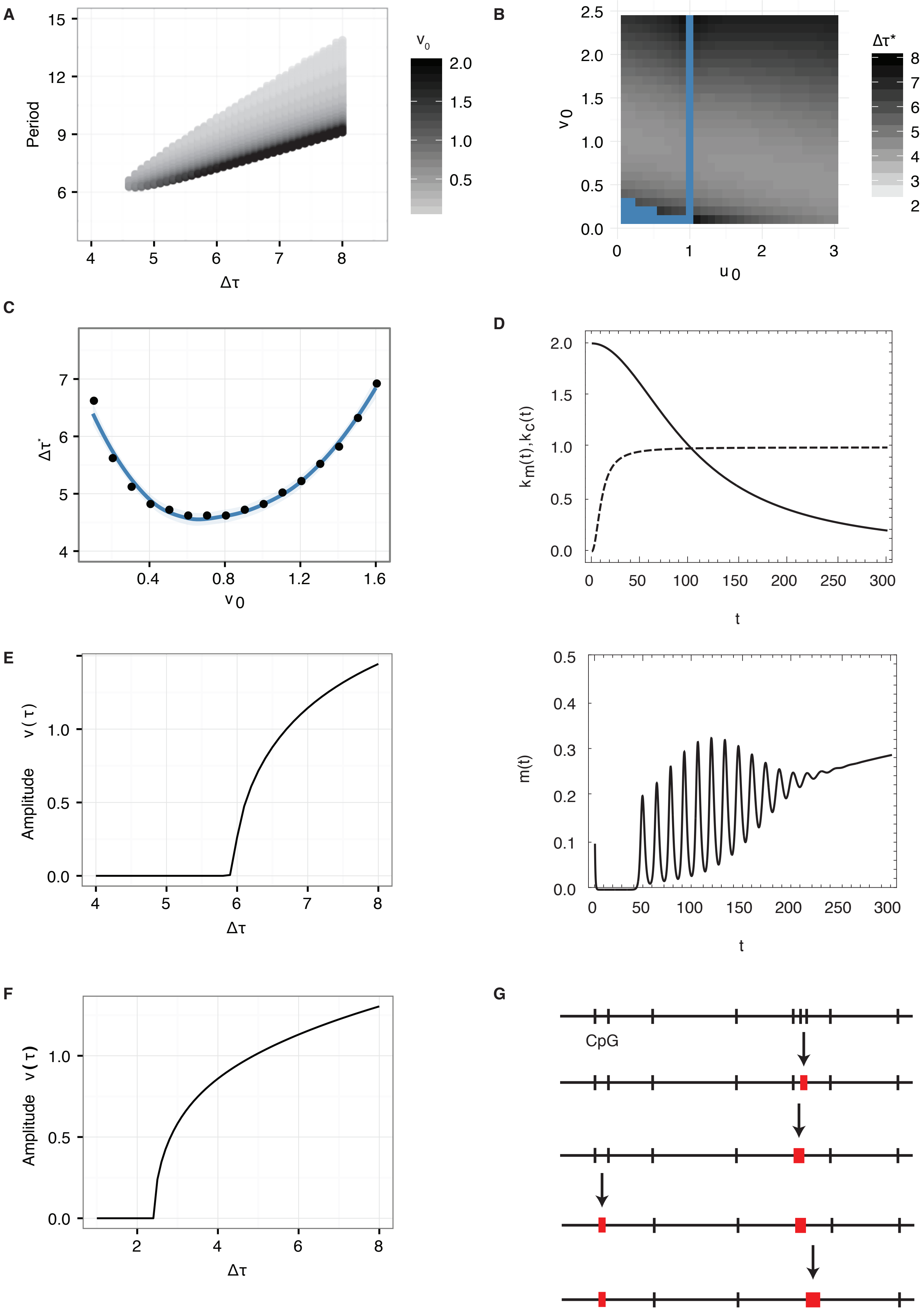

Figure S4. Related to Figure 3

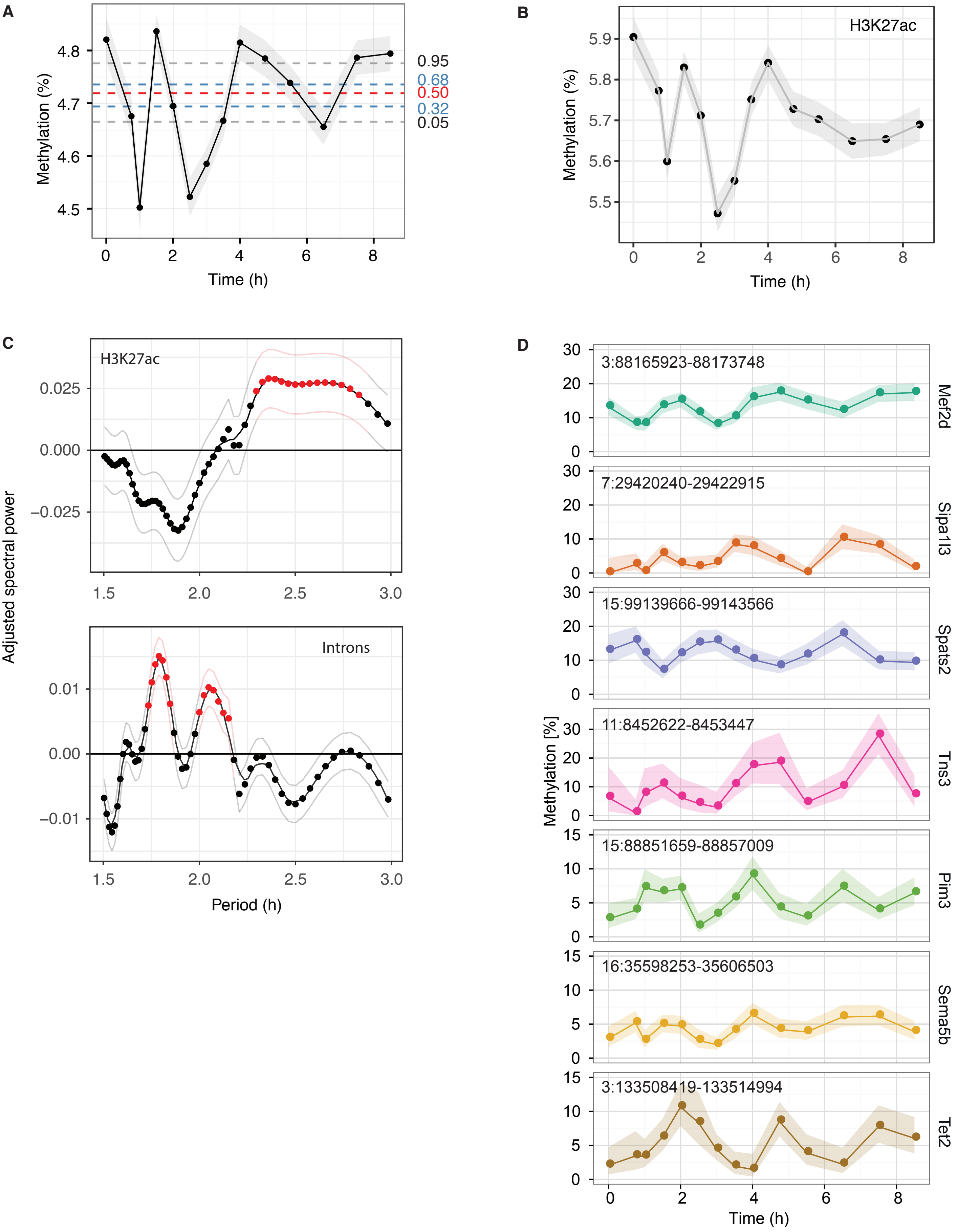

Figure S5. Related to Figure 3

A

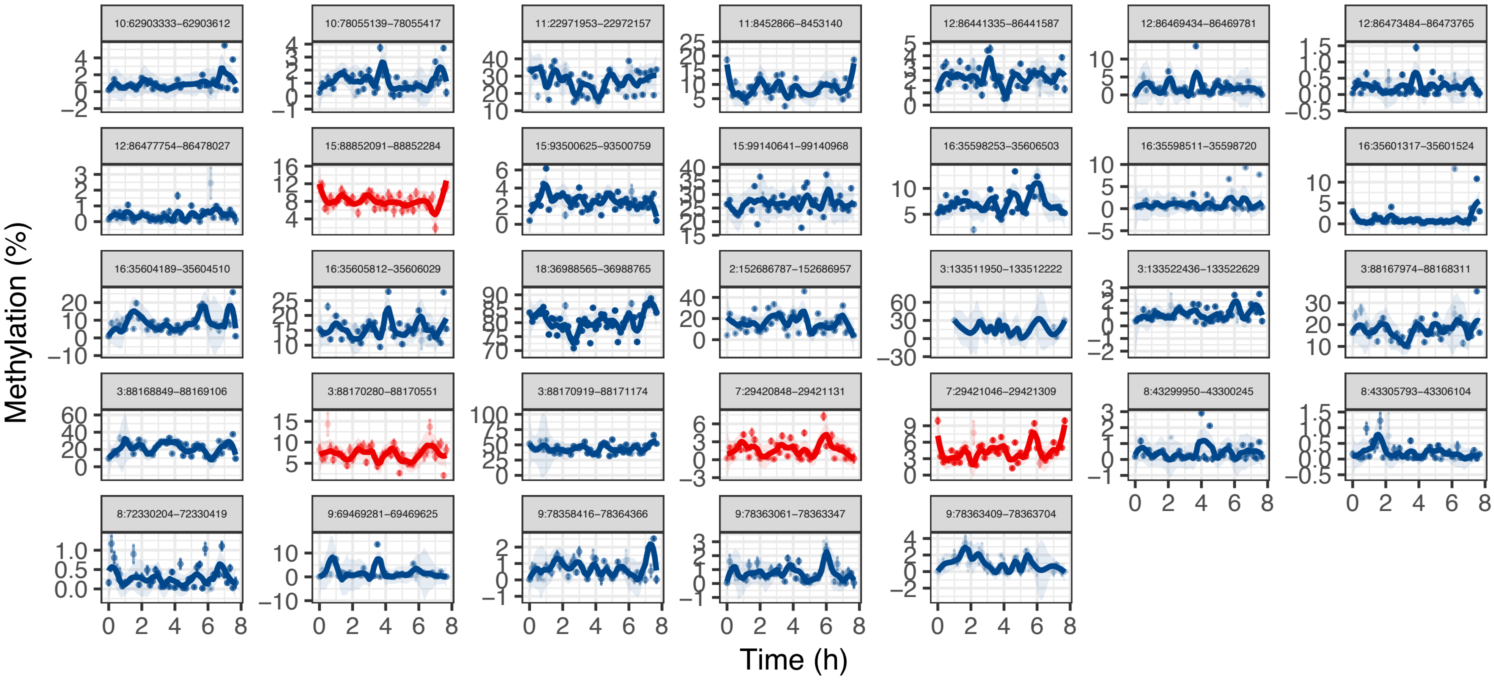

B

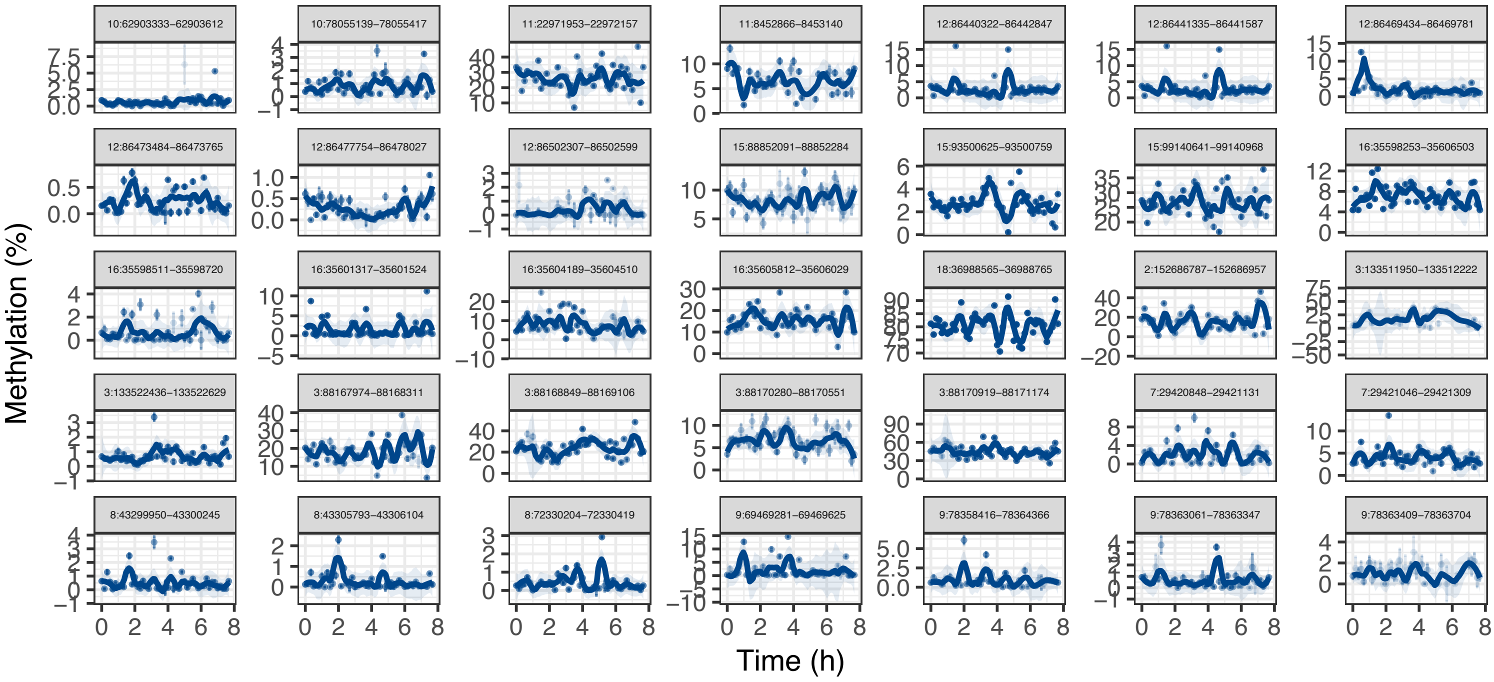

Figure S6. Related to Figure 5 and Figure 6

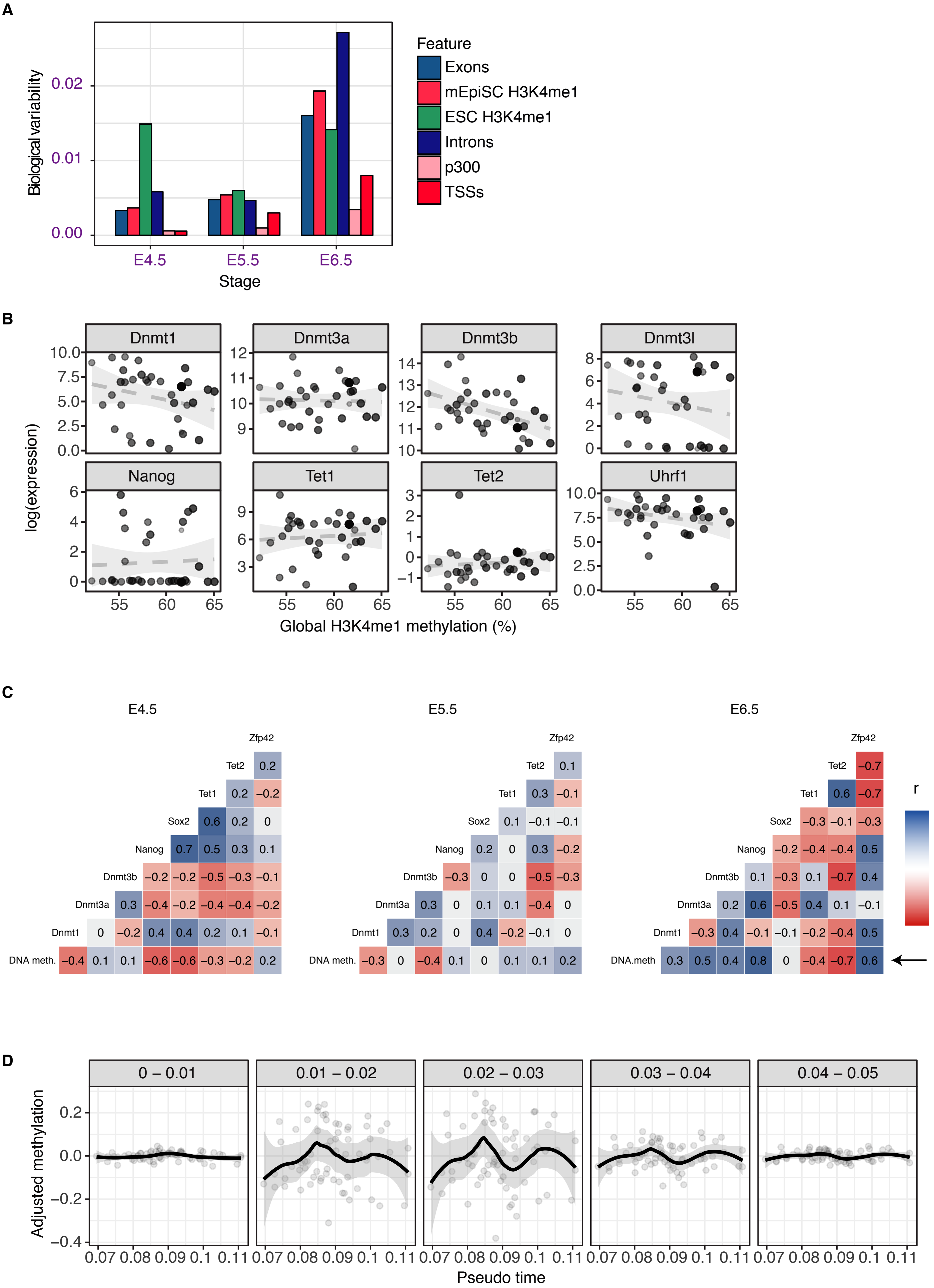

Table S1

Description	Reference
Primed ESC scM&T-seq	Angermueller et al (2016) Nature methods 13, 229-232
Primed ESC Dnmt3a/b ChIP-seq	Baubec et al (2015) Nature 520, 243-247
Naive ESC H3K4me1 ChIP-seq	Buecker et al (2014) Cell Stem Cell 14, 838-853
Primed ESC H3K27ac ChIP-seq	Creyghton et al (2010) Proceedings of the National Academy of Sciences 107, 21931-21936
Primed ESC H3K27me3 ChIP-seq	Creyghton et al (2010) Proceedings of the National Academy of Sciences 107, 21931-21936
Primed ESC H3K4me1 ChIP-seq	Creyghton et al (2010) Proceedings of the National Academy of Sciences 107, 21931-21936
Primed ESC H3K4me3 ChIP-seq	Creyghton et al (2010) Proceedings of the National Academy of Sciences 107, 21931-21936
Primed ESC p300 ChIP-seq	Creyghton et al (2010) Proceedings of the National Academy of Sciences 107, 21931-21936
EpiSC H3K4me1 ChIP-seq	Factor et al (2015) Cell Stem Cell 14, 854-863
Primed ESC replication timing	Hiratani et al (2010) Genome Research 20, 155-169
CpG islands (CGIs)	Illingworth et al (2010) PLoS Genetics 6, e1001134
E6.5 scRNA-seq	Sciadlone et al (2016) Nature 535, 289-293
Primed ESC mRNA half-life	Sharova et al (2009) DNA Research 16, 45-58
Naïve and primed ESC scBS-seq and BS-seq	Smallwood et al (2014) Nature methods 11, 817-820
Primed ESC low methylated regions (LMRs)	Stadler et al (2011) Nature 480, 490-495

Table S2

Gene ID	Gene Symbol
ENSMUSG00000031972	Acta1
ENSMUSG00000035783	Acta2
ENSMUSG00000015143	Actn1
ENSMUSG00000069833	Ahnak
ENSMUSG00000021057	Akap5
ENSMUSG00000013076	Amotl1
ENSMUSG00000024659	Anxa1
ENSMUSG00000032231	Anxa2
ENSMUSG00000029484	Anxa3
ENSMUSG00000092060	Bend4
ENSMUSG00000050071	Bex1
ENSMUSG00000020423	Btg2
ENSMUSG00000029761	Cald1
ENSMUSG00000000303	Cdh1
ENSMUSG00000023906	Cldn6
ENSMUSG00000001349	Cnn1
ENSMUSG00000004665	Cnn2
ENSMUSG00000032060	Cryab
ENSMUSG00000020661	Dnmt3a
ENSMUSG00000000730	Dnmt3l
ENSMUSG00000022048	Dpysl2
ENSMUSG00000015932	Dstn
ENSMUSG00000021255	Esrrb
ENSMUSG00000013089	Etv5
ENSMUSG00000028128	F3
ENSMUSG00000034391	Fbxo15
ENSMUSG00000050917	Fgf4
ENSMUSG00000028270	Gbp2
ENSMUSG00000030117	Gdf3
ENSMUSG00000028480	Glipr2
ENSMUSG00000026879	Gsn
ENSMUSG00000020644	Id2
ENSMUSG00000025950	Idh1
ENSMUSG00000038518	Jarid2
ENSMUSG00000055148	Klf2
ENSMUSG00000003032	Klf4
ENSMUSG00000023043	Krt18
ENSMUSG00000020911	Krt19
ENSMUSG00000023039	Krt7
ENSMUSG00000049382	Krt8
ENSMUSG00000021959	Lats2
ENSMUSG00000068220	Lgals1
ENSMUSG00000033306	Lpp
ENSMUSG00000036940	Lsd1
ENSMUSG00000031207	Msn
ENSMUSG00000022443	Myh9
ENSMUSG00000012396	Nanog

ENSMUSG00000025056	Nr0b1
ENSMUSG00000030770	Parva
ENSMUSG00000006494	Pdk1
ENSMUSG00000002265	Peg3
ENSMUSG00000021196	Pfkp
ENSMUSG00000033149	Phldb2
ENSMUSG00000021701	Plk2
ENSMUSG00000018217	Pmp22
ENSMUSG00000024406	Pou5f1
ENSMUSG00000036030	Prtg
ENSMUSG00000051176	Rex1
ENSMUSG00000041959	S100a10
ENSMUSG00000027907	S100a11
ENSMUSG00000001025	S100a6
ENSMUSG00000031665	Sall1
ENSMUSG00000027547	Sall4
ENSMUSG00000028645	Slc2a1
ENSMUSG00000003153	Slc2a3
ENSMUSG00000074637	Sox2
ENSMUSG00000038156	Spon1
ENSMUSG00000029304	Spp1
ENSMUSG00000046323	Stella
ENSMUSG00000032085	Tagln
ENSMUSG00000041359	Tcl1
ENSMUSG00000032494	Tdgf1
ENSMUSG00000021953	Tdh
ENSMUSG00000055320	Tead1
ENSMUSG00000047146	Tet1
ENSMUSG00000040943	Tet2
ENSMUSG00000026380	Tfcp2l1
ENSMUSG00000040152	Thbs1
ENSMUSG00000032366	Tpm1
ENSMUSG00000072235	Tuba1a
ENSMUSG00000001473	Tubb6
ENSMUSG00000020407	Upp1
ENSMUSG00000047751	Utf1
ENSMUSG00000042712	Wbp5
ENSMUSG00000028173	Wls
ENSMUSG00000038872	Zfhx3

Table S4

Gene	Comment	Forward Primer Sequence	Reverse Primer Sequence
Atp5b	Housekeeping control	GGCCAAGATGTCCTGCTGTT	GCTGGTAGCCTACAGCAGAAGG
Dnmt3a	*de novo* DNA methylase	CCTGCAATGACCTCTCCATT	CAGGAGGCGGTAGAACTCAA
Dnmt3b	*de novo* DNA methylase	TGGTGATTGGTGGAAGCC	AATGGACGGTTGTCGCC
Tet1	DNA methylation hydroxylase	CCATTCTCACAAGGACATTCACA	GCAGGACGTGGAGTTGTTCA
Tet2	DNA methylation hydroxylase	GCCATTCTCAGGAGTCACTGC	ACTTCTCGATTGTCTTCTCTATTGAGG

Table S5

Oligo Name	Sequence 5' to 3' (include modification codes if applicable)
PE1_D501	AATGATACGGCGACCACCGAGATCTACACTATAGCCTACAC TCTTTCCCTACACGACGCTCTTCCGATC*T
PE1_D502	AATGATACGGCGACCACCGAGATCTACACATAGAGGCACAC TCTTTCCCTACACGACGCTCTTCCGATC*T
PE1_D503	AATGATACGGCGACCACCGAGATCTACACCCTATCCTACAC TCTTTCCCTACACGACGCTCTTCCGATC*T
PE1_D504	AATGATACGGCGACCACCGAGATCTACACGGCTCTGAACAC TCTTTCCCTACACGACGCTCTTCCGATC*T
PE1_D505	AATGATACGGCGACCACCGAGATCTACACAGGCGAAGACAC TCTTTCCCTACACGACGCTCTTCCGATC*T
PE1_D506	AATGATACGGCGACCACCGAGATCTACACTAATCTTAACAC TCTTTCCCTACACGACGCTCTTCCGATC*T
PE1_D507	AATGATACGGCGACCACCGAGATCTACACCAGGACGTACAC TCTTTCCCTACACGACGCTCTTCCGATC*T
PE1_D508	AATGATACGGCGACCACCGAGATCTACACGTACTGACACAC TCTTTCCCTACACGACGCTCTTCCGATC*T
